# Untangling the hedge: Genetic diversity in clonally and sexually transmitted genomes of European wild roses, *Rosa* L.

**DOI:** 10.1371/journal.pone.0292634

**Published:** 2023-10-05

**Authors:** Katja Reichel, Veit Herklotz, Alisia Smolka, Hilde Nybom, Alexandra Kellner, Jan De Riek, Marinus J. M. Smulders, Volker Wissemann, Christiane M. Ritz

**Affiliations:** 1 Institute of Biology, Dahlem Center of Plant Sciences, Freie Universität Berlin, Berlin, Germany; 2 Department of Botany, Senckenberg Museum for Natural History Görlitz, Senckenberg–Member of the Leibniz Association, Görlitz, Germany; 3 Department of Plant Breeding, Balsgård, Swedish University of Agricultural Sciences, Kristianstad, Sweden; 4 Institute of Botany, Systematic Botany Group, Justus-Liebig-University, Gießen, Germany; 5 Flanders Research Institute for Agricultural, Fisheries and Food Research (ILVO), Plant Sciences Unit, Melle, Belgium; 6 Plant Breeding, Wageningen University & Research, Wageningen, The Netherlands; 7 International Institute (IHI) Zittau, Chair of Biodiversity of Higher Plants, Technical University Dresden, Zittau, Germany; University of Delhi, INDIA

## Abstract

While European wild roses are abundant and widely distributed, their morphological taxonomy is complicated and ambiguous. In particular, the polyploid *Rosa* section *Caninae* (dogroses) is characterised by its unusual meiosis, causing simultaneous clonal and sexual transmission of sub-genomes. This hemisexual reproduction, which often co-occurs with vegetative reproduction, defies the standard definition of species boundaries. We analysed seven highly polymorphic microsatellite loci, scored for over 2 600 *Rosa* samples of differing ploidy, collected across Europe within three independent research projects. Based on their morphology, these samples had been identified as belonging to 21 dogrose and five other native rose species. We quantified the degree of clonality within species and at individual sampling sites. We then compared the genetic structure within our data to current rose morpho-systematics and searched for hemisexually co-inherited sets of alleles at individual loci. We found considerably fewer copies of identical multi-locus genotypes in dogroses than in roses with regular meiosis, with some variation recorded among species. While clonality showed no detectable geographic pattern, some genotypes appeared to be more widespread. Microsatellite data confirmed the current classification of subsections, but they did not support most of the generally accepted dogrose microspecies. Under canina meiosis, we found co-inherited sets of alleles as expected, but could not distinguish between sexually and clonally inherited sub-genomes, with only some of the detected allele combinations being lineage-specific.

## Introduction

The triple combination of hybridization, polyploidy and clonal reproduction is a frequent phenomenon in plant evolution [1–3]. The precise circumstances that give rise to any of the three processes, or ensure their maintenance, are still widely debated, e.g. in [4–7]. For instance, in the European flora, the resulting species complexes found in the genera *Alchemilla* L., *Taraxacum* F.H. Wigg., *Pilosella* Hill, *Hieracium* L., *Rubus* L. or *Rosa* L. remain a challenge to taxonomy [8]. Case-specific differences between individual species complexes, such as the unique meiosis in *Rosa* sect. *Caninae*, may provide a key to understanding the general mechanisms involved.

The genus *Rosa* is mainly distributed throughout temperate regions of the northern hemisphere, where it has undergone several polyploidization and hybridization events [9–11]. Diploid species are usually obligately outcrossing whereas polyploids can produce seeds after both outcrossing and selfing. In addition, many species reproduce vegetatively by root suckers. There are currently around 200 species within the *Rosa* genus, most of which belong to either of two clades: section *Synstylae* and its allies, or section *Rosa* and its allies [9, 12]. In contrast to North America and Asia, where other lineages have diversified, the European rose flora is dominated by the section *Caninae*, which belongs to the *Synstylae* clade. Species in section *Caninae*, commonly called dogroses, are typically pentaploid (i.e. possessing 2n = 5x = 35 chromosomes), but tetra-, hexa- and heptaploids also exist [13–20].

Dogrose systematics are traditionally based on morphology. There are three well-defined and genetically supported subsections within the *Rosa* section *Caninae* [21]: the subsection *Caninae*, within which hooked prickles, eglandular leaves or odourless glands prevail; the subsection *Rubigineae*, with hooked prickles and leaves with apple-scented glands; and the subsection *Vestitae*, with slightly curved or straight prickles, densely pubescent leaves and resin-scented glands. In each subsection, microspecies have been described based on a set of correlated characters according to the L/D-system [22–24]: In L-type species, a lax growth habit is combined with long pedicels, deflexed and deciduous sepals, a narrow diameter of the rose hip orifice and fruits ripening late in the season. In contrast, D-type species have a dense growth habit, short pedicels, upright persistent sepals, a wide diameter of the rose hip orifice and early maturing fruits. Intermediate forms (L/D-types) exist in each subsection and have also been given species status. However, some of these microspecies have been formed by polytopic hybridization [13, 25–27].

Beside vegetative and occasionally apomictic clonal reproduction [28, 29], the members of the section *Caninae* have evolved a unique form of meiosis, known as canina meiosis [30–33], which enables them to overcome the sexual sterility usually caused by uneven ploidy levels. During canina meiosis, only two sets of chromosomes form pairs (bivalents) in metaphase I, while all other chromosome sets remain unpaired (univalents). This results in two, three or four univalent chromosome sets occurring in tetraploids, pentaploids and hexaploids, respectively. DNA from the same nucleus is thus either passed on sexually (subject to mutation and recombination), when on a bivalent-forming chromosome, or clonally (subject to mutation only), when part of a univalent. In this hemisexual reproductive mode, the transmitted amount of DNA also differs between the sexes: only one set of the bivalent-forming chromosomes is enclosed in the pollen grain, whereas egg cells contain one set of the bivalent-forming chromosomes and all of the univalents. The role of each chromosome set as either bivalent or univalent is fixed [28, 34, 35]. Consequently, dogroses offer a unique study system for making direct comparisons between the evolution of sexually and clonally transmitted genomes in the same organismal and cellular environment.

Alleles observed at microsatellite or nuclear single-copy gene loci on bivalent chromosomes have so far been found to be highly homozygous [13, 28, 34, 35], with the number of different homologous alleles observed in an individual rarely exceeding its ploidy level minus one (i.e. four alleles in a pentaploid individual). Thus, allelic variation occurs mainly among clonally inherited univalent-forming genomes. This lends credence to the hypothesis that the described subsections within dogroses correspond to different evolutionary lineages, each characterized by having its own fixed set of univalent sub-genomes [34]. In contrast, cytogenetic studies have shown that the bivalent-forming chromosome sets differ across the *Caninae* subsections: sub-genomes that form bivalents in the subsection *Caninae* behave as univalents in the subsection *Rubigineae*, and *vice versa* [36–38]. When considered alongside plastid phylogenetic data [12, 39, 40], this supports the hypothesis that the canina meiosis originated independently on at least two occasions.

Resolving species delimitations in dogroses using genetic data has proved difficult due to their unusual reproductive mode [21, 41], and because the studies tend to cover only a restricted set of taxa and/or minor parts of the species’ distribution areas [13, 14, 25, 28, 34, 35, 42–44]. Studying codominant genetic marker diversity in data from broader taxonomic and geographic sampling efforts could therefore help test more specific hypotheses on dogrose evolution.

In our study, we use microsatellite allelic compositions of European wild roses belonging to different species, compiled from different studies across Europe, to address the following questions: 1) Does the level of clonal reproduction differ between dogrose species and other native rose species, or in relation to geography? 2) Does genetic structure at the European scale reflect the current morphology-based taxonomy? 3) Are univalent sub-genomes transmitted as sets, and if so, are these sets lineage-specific?

## Material & methods

### Sampling and study sites

**Fig 1:**
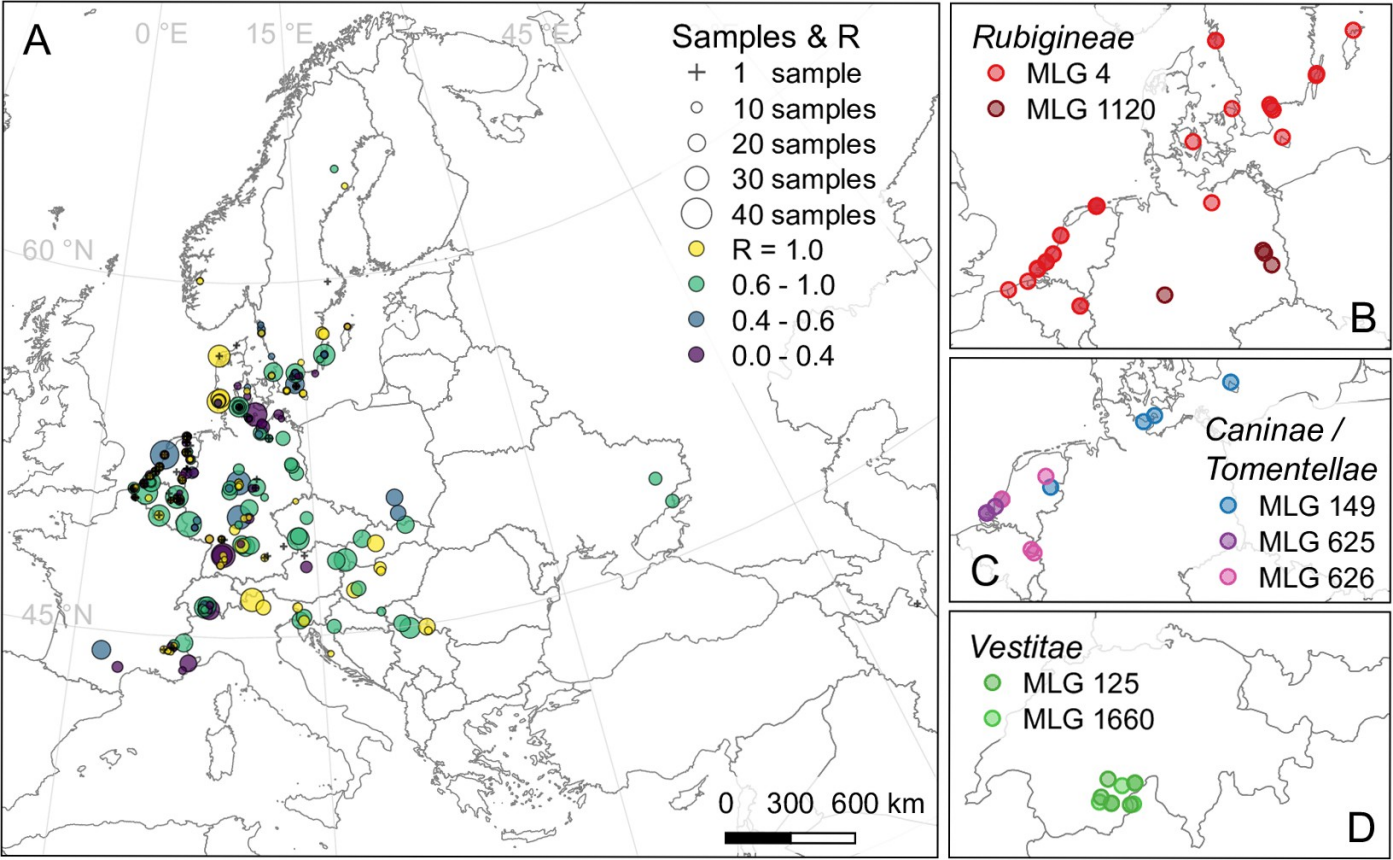
Map of sampling sites and geographic distribution of MLGs. (A) Map of sampling sites, showing number of samples (dot radius) and per-site clonal richness R (shade; all plants with distinct MLG at R = 1.00). (B-D). Spatial distribution of select MLGs shared between at least four sampling sites within a wider area. Samples with the same MLG were sometimes identified as different species, usually from the same subsection. Those shown here were identified as *R*. *rubiginosa* (MLG 4, 1120), *R*. *gremlii* (MLG 4, 1120) and *R*. *micrantha* (MLG 4)–*R*. *corymbifera* (MLG 149, 625, 626), *R*. *canina* (MLG 149) and *R*. *balsamica* (MLG 625, 626)–*R*. *villosa* (MLG 125, 1660). Albers equal area projection, country outlines from Natural Earth.

We analysed data from 2 615 native wild rose plants from 367 sites across Europe (Fig 1), belonging to 26 species with a particular focus on the section *Caninae*, which 21 of the species represent. Our data were compiled from three different data sets GE, HR and KW (Table 1, S1 File), the latter two of which have already been published on [13, 14, 25]. The largest data set (GE) originates from the Generose project [45], and has not been published on before. Across all three data sets, the number of samples collected per site ranged from 1 to 37, with a mean of 7.1 and a median of 4. Sufficient distance was kept between sampled individuals to prevent collecting suckers, especially in species with prevalent root suckering, such as *R*. *spinosissima* or *R*. *gallica*. Species were determined following the identification keys in [46] and/or by experts from the respective countries.

**Table 1:** Summary of sampled wild rose species originating from three different data sets.

Taxonomy			Data source			Samples		MLGs		Ploidy level estimate				
Section	Subsection	Species	KW	GE	HR	total	sites	G	sG	7x	6x	5x	4x	3x/2x
*Caninae*	*Caninae*	*R*. *caesia*		14		14	7	12	12			14		
		*R*. *canina*		523	313	836	107	642	653	4	134	698		
		*R*. *corymbifera*		95	87	182	61	144	144	2	38	142		
		*R*. *dumalis*		47	3	50	19	41	41		4	46		
		*R*. *stylosa*		3		3	2	2	2		3			
		*R*. *subcanina*		5	44	49	33	49	49		7	42		
		*R*. *subcollina*		11	10	21	11	16	17		5	16		
	*Rubigineae*	*R*. *agrestis*		13	63	76	21	53	56	5	29	42		
		*R*. *elliptica*		5		5	2	5	5		2	3		
		*R*. *gremlii*		39	87	126	35	72	87		16	110		
		*R*. *inodora*		8	24	32	11	23	24	2	16	14		
		*R*. *micrantha*		24	82	106	32	82	86	2	73	31		
		*R*. *rubiginosa*		106	25	131	47	59	84	1	15	115		
	*Rubrifoliae*	*R*. *glauca*		8		8	3	7	7			5	3	
	*Tomentellae*	*R*. *balsamica*		47		47	12	30	35		16	31		
	*Trachyphyllae*	*R*. *marginata*		10		10	2	8	8	2	5	3		
	*Vestitae*	*R*. *mollis*	63	22		85	11	67	68		1	41	43	
		*R*. *pseudo-scabriuscula*		5		5	2	2	2			5		
		*R*. *sherardii*	30	23		53	12	21	22		1	41	11	
		*R*. *tomentosa*		53		53	10	29	30		11	42		
		*R*. *villosa*	99	71		170	23	82	111		11	47	112	
*Gallicanae*		*R*. *gallica*		136		136	13	37	42				109	27^a^
*Pimpinellifoliae*		*R*. *spinosissima*		296		296	28	211	224				200	96^b^
*Rosa*		*R*. *majalis*		20		20	6	13	13					20
		*R*. *pendulina*		10		10	2	4	4				5	5^c^
*Synstylae*		*R*. *arvensis*		91		91	13	49	55					91
** **		**Total**	**192**	**1685**	**738**	**2615**	**378**	**1715**	**1881**	**18**	**387**	**1488**	**483**	**239**

Data source: KW [14], HK [13] and GE (new data, project described in [45]). Samples: total number per species, number of sites from which species was sampled. MLGs, distinct multilocus genotypes: number per species (i.e. genotypic richness G), number per species if identical MLGs from different sampling sites were counted as distinct (sG). Ploidy level estimate: number of samples per ploidy level (see Methods).

^a^ Includes 23 potentially triploid samples. ^b^ Includes 94 samples scored as triploid. ^c^ All scored as triploid.

### DNA extraction and microsatellite analyses

Genomic DNA was extracted from silica gel-dried leaf samples, quantified and stored at -80°C until use. For all samples, we performed PCRs to retrieve allele combinations at seven microsatellite loci, originally developed by [47]: loci *RhAB73*, *RhP50* and *RhP518* each belong to different linkage groups, whereas the pairs of loci *RhO517* and *RhD201*, as well as *RhEO506* and *RhB303*, each share a linkage group. Details on DNA extraction, PCR protocols, conditions of amplification and fragment sizing for the GE, HR and KW data sets are given in the respective publications [13, 14, 25, 45, 48], and presented in overview in S3 File.

Since allele dosage of polyploids can barely be assessed from microsatellite data, we report on allelic phenotypes (hereafter, for simplicity, “genotypes”). Absolute fragment lengths can differ slightly between laboratories due to e.g. different sequencing machines and size standards. Based on a comparison of the most frequent fragments in the three data sets, we assumed a linear size shift and adjusted each locus, where required, by adding or subtracting of a maximum of 3 bp to all fragments. A PCoA of our final dataset revealed no data source specific effects.

### Ploidy estimation

Ploidy levels were determined in ~ 930 samples using Flow Cytometry for the KW and HR data sets, see [13, 14]. For the remaining samples, we estimated ploidy from the maximum number of distinct alleles across all loci in a given sample for the sections *Rosa*, *Gallicanae*, *Pimpinellifoliae* and *Synstylae*. In *R*. *gallica*, *R*. *spinosissima* and *R*. *pendulina*, this resulted in some samples that scored less than the 4x (or 3x) ploidy which had elsewhere been determined from Flow Cytometry and chromosome counts for these species [49, 50]. For individuals of the section *Caninae*, ploidy was instead estimated from the maximum number of alleles plus one, since previous studies showed that members of this section typically have at least one allele with two copies [28, 34, 35]. Ploidy level estimates are presented in Table 1 and in S1 File.

### Multilocus genotypes

We generated unique index numbers for all distinct sets of alleles detected across all loci within the same sample, i.e. for each distinct multilocus genotype (MLG). Since some samples from different species or sites had identical MLGs (see Fig 1 and S2 File), we also generated a second index, which treats identical MLGs from different sites or species as distinct. Both indices are included in S1 File. Depending on the properties and assumptions of further analyses, we either included data for all samples, excluded only copies of MLGs from the same species and site, or kept only one MLG copy per species.

For both MLG indices, we calculated the observed number of distinct MLGs (Table 1), i.e. genotypic richness (G), per species. Based on our first MLG index, we calculated clonal richness as R = (G-1) / (N-1) according to [51] per site, and displayed the values in a map (Fig 1), together with the distribution of select MLGs that were found at four or more sites across a wider area. Based on our second MLG index, we calculated clonal richness (R), Shannon’s diversity index (H) and Evenness (E) for each species in the dataset. The results for the latter two are included in S2 File. Both indexing and analyses were performed with a dedicated script written in Python 3.7.0, using the module *pandas 0*.*25*.*1* [52, 53], with maps and plots drawn with QGIS 3.10.9 [54] or in R 4.1.2 [55] using *ggplot2* [56].

### Genetic structure

To assess genetic structure between taxonomic entities, we performed Principal Coordinate Analyses (PCoA) using the R package *ape 5*.*0* [57], based on Bruvo mean distances between genotypes [58] with *polysat* [59], and retaining one copy of each MLG per species. Bruvo distances are based on a stepwise mutation model of microsatellite evolution, however, both this model and its application for measuring genetic distances have been heavily criticized, e.g. in [60–62]. In spite of this, we used the Bruvo distances in our study as they are well suited for polyploid samples [63], the data are thus compatible with previous publications [13, 25], and because, in our case, the PCoA plots based on other genetic distance measures, according to [63], yielded highly similar results (not shown). All plots were drawn in R 4.2.2 [55] using *ggplot2* [56]. To estimate the amount of variance between different taxonomic levels of roses we performed Analyses of Molecular Variance based on Bruvo distances and 999 permutations with the R package *poppr 2*.*9*.*3* [64, 65].

### Most frequent allele combinations in the section *Caninae*

If subsections within the section *Caninae* originated from different hybridization events, we would expect microsatellite alleles on the maternally inherited univalent genomes to be transmitted clonally as a fixed combination identical to the ancestral state, with occasional mutations being the only source of diversity. In contrast, alleles on the two bivalent-forming genomes would be able both to mutate and to be exchanged through recombination. Previous studies [28, 34, 35] have shown that the bivalent alleles tend to be highly homozygous within individuals and rather homogeneous within each subsection. Rather than random combinations of five (or four) alleles per locus in a pentaploid (or tetraploid) dogrose sample, we would thus expect to see combinations of four (in pentaploids) or three (in tetraploids) alleles that are:

- very common across samples (“high frequency”)
- linked to decreasingly frequent variant combinations by an exchange of one (or, less frequently, more) of the included alleles (“high stability”), and
- potentially characteristic for each subsection (“high specificity”).

Depending on their overall frequency, which is influenced by the per-locus mutation rate, these characteristic combinations need not be evident directly from the observed frequencies of single alleles. Moreover, the same allele length may represent different homologous copies of the locus (“hidden” allele; typical for bivalent alleles but also possible in univalent alleles). Null alleles (usually caused by mutations in primer binding sites), the mutational loss of a locus in one sub-genome, or the loss of an entire ancestral sub-genome (e.g. as possibly in 4x *Vestitae*), may lead to additional deviations from the expected outcome.

To test our hypothesis, for each locus and in each of the three major subsections of the section *Caninae*, i.e. *Caninae*, *Rubigineae* and *Vestitae*, we calculated:

- the apparent number of alleles per sample (S2 File),
- allele prevalence (proportion of samples with the allele, S2 File) and relative allele frequency (apparent proportion of the allele among all detected alleles) for each allele, and
- the frequencies of the ten most frequent combinations of four, three or two alleles.

Samples from subsection *Vestitae* were further split into two groups according to their estimated ploidy (4x and 5x). In general, only samples that appeared to be tetra- or pentaploid were used for this analysis, since hexaploids often appear to originate from hybridization between subsections [13]. Although the calculated allele frequencies are not exact, due to null or hidden alleles, they constitute the best approximation of the “real” allele frequencies available for statistical tests. To check for a potential effect of clonal amplification of individual MLGs, we conducted the analysis with and without repeat MLGs from the same species and site, but we found only minimal differences (S2 File). We therefore included all available data in further calculations.

**Fig 2:**
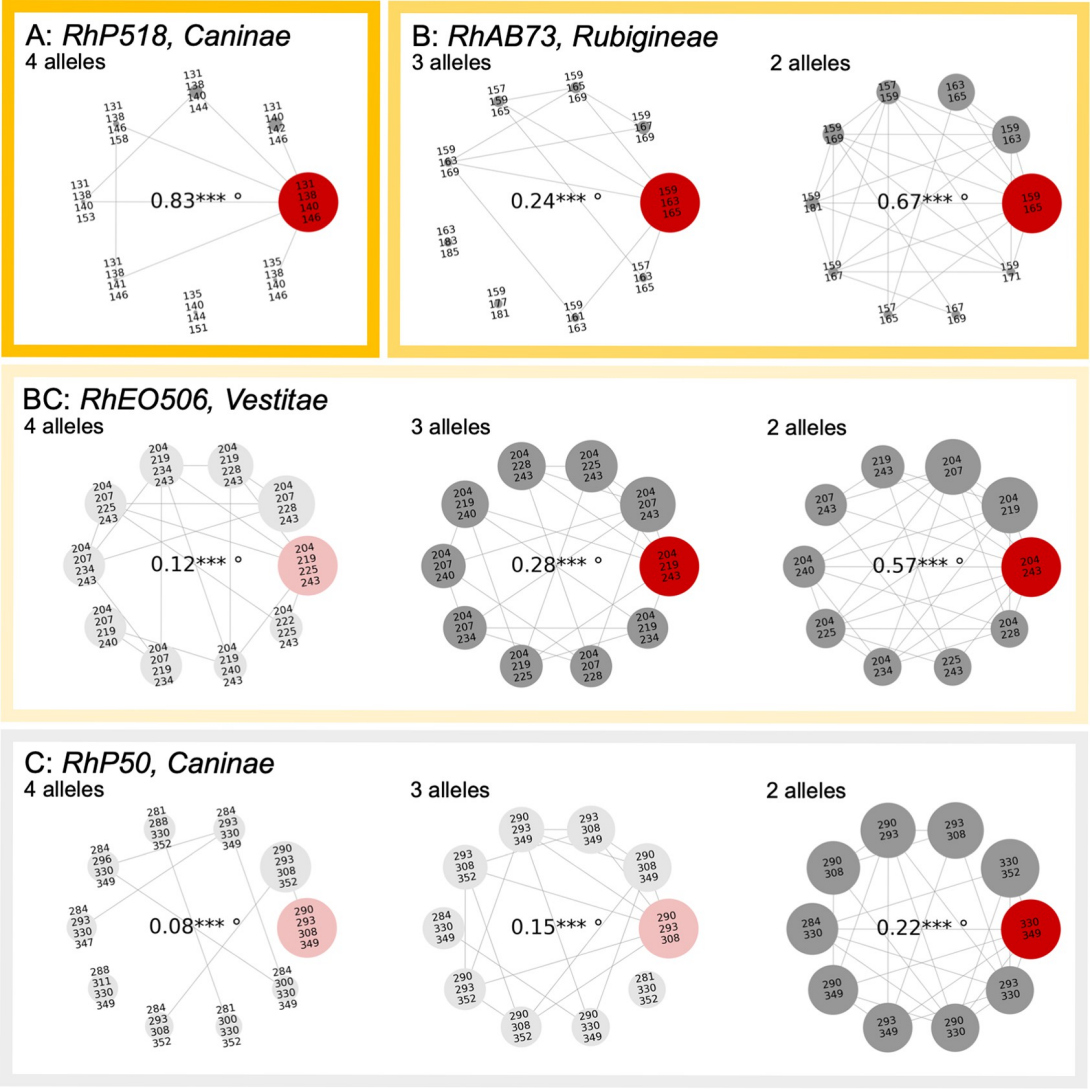
Examples of network plots for loci sorted into categories A, B, BC and C (see text). Allele combinations differing by only one allele are connected by lines. Circle diameter corresponds to the frequency of the respective allele combination, relative to the most frequent combination (MFAC, in red, maximum diameter). The frequency of the MFAC is displayed in the centre of the network, marked by stars if significantly higher than random (* p ≤ 0.05, ** p ≤ 0.01, *** p ≤ 0.001), and additionally by a degree sign if significantly elevated within the respective subsection (° p ≤ 0.001).

To display our results, we designed special network plots (examples in Fig 2, all plots in S2 File), within which nodes correspond to the up-to-ten most frequent allele combinations, ordered counter-clockwise with decreasing frequency. Connecting lines are drawn only between nodes / allele combinations that differ by exactly one allele, irrespective of the associated repeat copy number, i.e. without assuming a stepwise mutation model. Node size corresponds to the frequency of each allele combination relative to the absolute frequency of the most frequent combination, as displayed in the centre of the plot. If this frequency is below 0.2, the plot is faded to provide a better overview.

To evaluate the high frequency criterion, we used a one-sided binomial significance test, which verifies that each of the most frequent allele combinations (MFAC) was more frequent than expected from randomly drawing alleles from the estimated distribution of allele frequencies for the respective locus and group, taking the possibility of multiple same-length alleles into account (i.e. comparable to the frequency of each allele combination expected at Hardy-Weinberg equilibrium under completely sexual penta-/tetrasomic inheritance). To test for high specificity, we compared MFACs between subsections and used a one-sided hypergeometric test to see if the MFAC was significantly more frequent in the respective partial dataset than in the mean across all groups, weighted to account for the different numbers of samples. Both high frequency (stars) and high specificity (degree sign) are marked in the centre of the network plots (Fig 2).

To assess the high stability criterion, we visually compared the network plots for four (in pentaploids), three and two alleles at each locus within each group. Ideally, the MFAC with the maximum number of alleles should contain all the most frequent allele combinations with fewer alleles. Due to the occasional mutation of single alleles (as illustrated by the connecting lines within networks when no apparent allele was lost), “allele subsets” should, however, be more frequent than the “complete” MFAC. Comparing both the MFAC frequencies and the actual alleles involved across MFACs for different numbers of alleles served as a plausibility check, and it allowed us to identify highly frequent allele combinations with potential hidden or null alleles (especially at loci with an apparent number of alleles below four in pentaploids, or below three in tetraploids). Based on these comparisons, we sorted all locus-group combinations into four different categories A, B, BC and C (Fig 2), which are further explained in the Results.

## Results

### Multilocus genotypes and clonal richness

Descriptive statistics of our data, including ploidy levels and the taxonomic and geographic distribution of wide-spread repeat MLGs, are given in Table 1 and Fig 1. A map of clonal richness at different sampling sites (disregarding species classification; Fig 1) showed no clear geographic pattern. According to expectations, most shared mutilocus genotypes (MLGs) belong to the same species (S2 File). However, we also found the same MLG in different microspecies growing on the same site (especially in subsection *Rubigineae*), and even a few cases where MLGs were shared between species from different subsections. The first observation reflects the difficulty in accurately identifying microspecies; the latter is probably a result of too low resolution of the studied microsatellite markers, imprecise genotyping due to null alleles, or a different allele being present in duplicate in samples showing the same MLG.

Overall, 79 MLGs were detected at more than one sampling site, and only 16 at more than two. While sites sharing MLGs were often in close proximity, such as four sites on the same West Frisian Island with six samples (all MLG 599) assigned to three microspecies from the subsection *Caninae*, this was not a general rule. Remarkably, one genotype (MLG 4; Fig 1B) shared between three microspecies from the subsection *Rubigineae* was found at 29 sites up to more than 1 000 km apart, ranging from the west Belgian coast to Gotland island (Sweden). A second genotype (MLG 149; Fig 1C) shared between two microspecies from the subsection *Caninae* had a similar, albeit smaller, geographic distribution. The greatest distance measured between pairs of samples with an identical MLG was over 1 600 km (S2 File). Other MLGs shared between sites appeared to be more locally common, e.g. across the Netherlands or in parts of Switzerland (Fig 1C and 1D).

**Fig 3:**
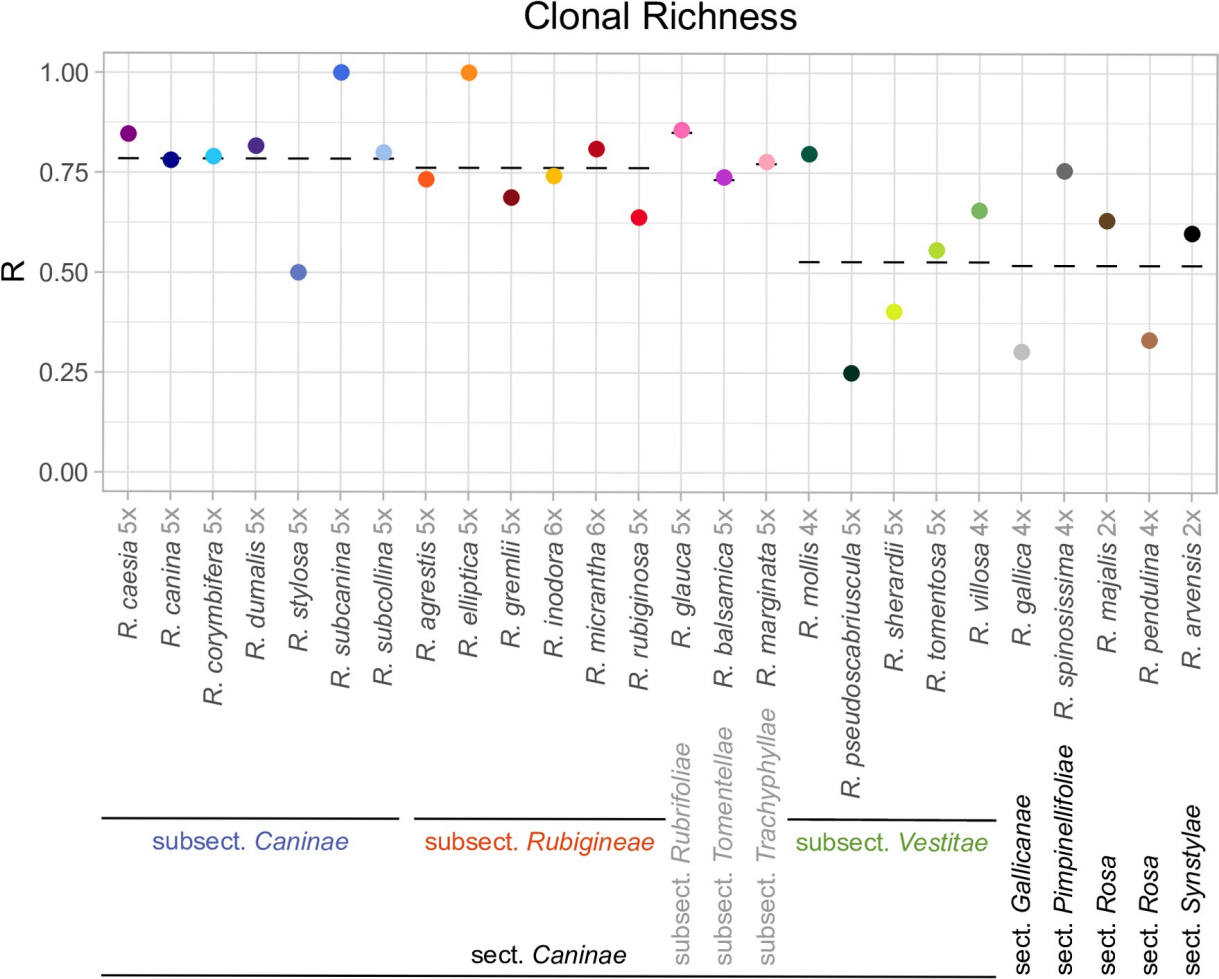
Clonal richness R of each investigated species. 0 = all plants identical, 1 = all plants unique. Dashes denote mean values per subsection in section *Caninae*, or the mean across all other sections. Ploidy levels after species names refer to the majority of our samples (see Table 1).

Apart from the subsection *Vestitae*, species of the section *Caninae* showed higher R values, and thus a lower proportion of repeat MLGs, compared to roses with regular meiosis (Fig 3). Species of the subsection *Caninae* were slightly more diverse than those of the subsection *Rubigineae*. All analysed samples from the microspecies *R*. *elliptica* (subsection *Rubigineae;* represented by five individuals from two sites), as well as from *R*. *subcanina* (subsection *Caninae;* represented by 49 samples from 33 sites; see Table 1), were genetically distinct. *Rosa stylosa* (subsection *Caninae*) and *R*. *pseudoscabriuscula* (subsection *Vestitae*) had a much lower MLG to samples ratio than other species from their respective subsections. Within the subsection *Vestitae*, R values were not correlated with the predominant ploidy level of the samples. Among roses with regular meiosis, the tetraploid *R*. *spinosissima* (section *Pimpinellifoliae*) was much more diverse compared to the other tetraploid species *R*. *gallica* (section *Gallicanae*) and *R*. *pendulina* (section *Rosa*).

### Genetic differentiation between taxonomic units in wild roses

**Fig 4:**
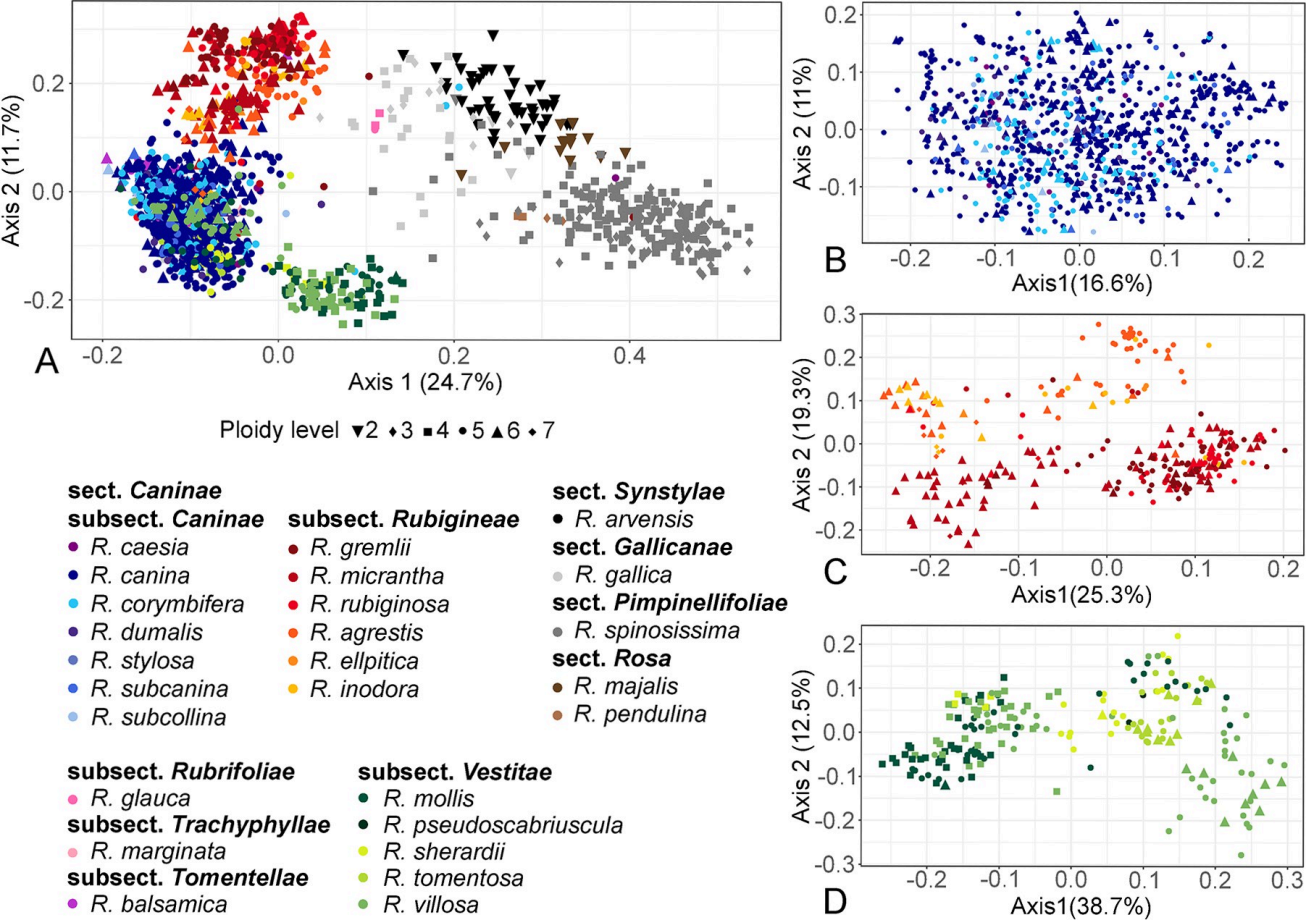
Principal coordinate analyses of all investigated samples. One copy per MLG per species, based on a total of 236 alleles at seven microsatellite loci. Colours indicate species/subsections; symbols indicate estimated ploidy level. (A) all taxonomic units; (B) subsection *Caninae* only; (C) subsection *Rubigineae* only; (D) subsection *Vestitae* only.

The PCoA of all samples separated the hemisexual section *Caninae* from all other rose sections along the first axis (Fig 4A), which explained more than 24% of the variation. Only samples of *R*. *gallica* (section *Gallicanae*) were situated in an intermediate position. In addition, the few samples (seven MLGs) of *R*. *glauca* (section *Caninae*, subsection *Rubrifoliae*) were separated from the remaining *Caninae* along the first axis. Samples from roses with regular meiosis were shown in two connected clusters of either *R*. *arvensis* (section *Synstylae*) or *R*. *spinosissima* (section *Pimpinellifoliae*), with samples from the section *Rosa* (*R*. *majalis*, *R*. *pendulina*) clustering at the junction between them.

Within the section *Caninae*, three groups corresponding to the subsections *Rubigineae* (red), *Caninae* (blue) and *Vestitae* (green) can be recognized mainly along the second axis. Approximately half of the samples belonging to the subsection *Vestitae* were shown intermingled with those of the subsection *Caninae*. The eight MLGs of *R*. *marginata* (light pink; subsect. *Trachyphyllae*) were intermingled with the subsection *Caninae* cluster, with some being located at its periphery towards the *R*. *gallica* samples. In addition, samples of *R*. *balsamica* (violet; subsection *Tomentellae*) clustered in the upper part of the subsection *Caninae* cluster, close to the samples from the subsection *Rubigineae*.

The PCoA of the subsection *Caninae* (Fig 4B) showed no structure: neither ploidy levels nor microspecies were separated. The majority of samples belonged to *R*. *canina* and *R*. *corymbifera*, which were both scattered across the entire plot. The PCoA of the subsection *Rubiginae* (Fig 4C) showed a weak differentiation between the *R*. *rubiginosa* agg. (rounded leaflet base, glandular pedicels: *R*. *rubiginosa*, *R*. *gremlii*, *R*. *micrantha*) and the *R*. *inodora* agg. (cuneate leaflet base, eglandular pedicels: *R*. *elliptica*, *R*. *inodora*, *R*. *agrestis*) along the second axis. However, samples did not cluster according to microspecies within the aggregates. Most of the hexaploid or even presumably heptaploid samples were clustered on the left side of the diagram. Samples from the subsection *Vestitae* (Fig 4D) were partially clustered by ploidy level: all tetraploid samples of *R*. *mollis* and *R*. *villosa* (i.e. *R*. *villosa* agg.) clustered on the left side, whereas the pentaploid and presumably hexaploid samples of these species either clustered with the tetraploids or formed a loose second cluster on the right side. Microspecies of *R*. *tomentosa* agg. (*R*. *tomentosa*, *R*. *pseudoscabriuscula* and *R*. *sherardii*) had an intermediate position between species of *R*. *villosa* agg.

Corresponding to the results of PCoAs at different taxonomic levels, AMOVA (Table 2) revealed considerable differentiation between sections (22.4%) and *Caninae* subsections (15%; subsections *Caninae*, *Rubigineae* and *Vestitae* only). Only 2.2% of the total variance was attributed to differences between microspecies of the subsection *Caninae*, which is much lower than the 14.9% and 18.1% that were detected between microspecies of the subsections *Rubigineae* and *Vestitae*, respectively.

**Table 2:** Results of AMOVA analyses regarding different taxonomic levels within European roses.

Source of variation	df	Sum of squares	Mean of squares	% variance	Φst-value
Between sections^1^	4	106.92	26.73	22.40	0.22
Between species within sections	21	94.20	4.49	11.56	0.15
Within species	1734	747.75	0.43	66.03	0.34
Between subsections of *Caninae*	2	57.13	28.57	15.01	0.15
Between species within subsections of *Caninae*^2^	15	25.22	1.68	5.48	0.06
Within species	1383	501.28	0.36	79.52	0.20
Between microspecies of the subsection *Caninae*	6	4.98	0.83	2.19	0.02
Within microspecies	899	289.55	0.32	97.81	
Between microspecies of the subsection *Rubigineae*	5	7.11	1.42	14.93	0.15
Within microspecies	288	45.08	0.16	85.07	
Between aggregates of the subsection *Rubigineae*	1	4.29	4.29	17.67	0.18
Within aggregates	292	47.89	0.16	82.33	
Between microspecies of the subsection *Vestitae*	4	5.31	1.33	18.13	0.18
Within microspecies	196	29.97	0.15	81.87	

^1^between the sections *Caninae*, *Gallicanae*, *Pimpinellifoliae*, *Rosa*, *Synstylae*

^2^subsections *Caninae*, *Rubigineae* and *Vestitae* only (subsections *Tomentellae*, *Rubrifoliae* and *Trachyphyllae* omitted)

### Frequent allele combinations in section *Caninae*

Patterns of allele diversity are clearly distinct at the seven studied microsatellite loci, and differ between subsections for some loci. This is already evident in the data for allele prevalence, as well as for the apparent number of alleles per locus and sample (S2 File). The overall number of distinct alleles detected per locus, i.e. allelic richness, ranged from 17 alleles in *RhO517* to 70 alleles in *RhP50*. The median apparent number of alleles found at the same locus within a sample, ranging from two to ploidy minus one, was often different between subsections. However, only in two of the three most polymorphic loci, i.e. *RhE506* and *RhP50*, did samples regularly display one allele less than their ploidy level. At the other loci, the number of different alleles per sample was usually lower. A few loci contained alleles that were unique for a subsection (e.g. 222 and 231 in *RhE506*, and 169 and 225 in *RhD201* for the subsection *Rubigineae*; 195 in *RhE506* and 180 in *RhD201* for the subsection *Caninae*), however, alleles that were frequently present across all subsections were found at most loci (e.g., 204 and 243 in *RhE506*; 194 and 202 in *RhD201*; 131 and 138 in *RhP518*).

**Fig 5:**
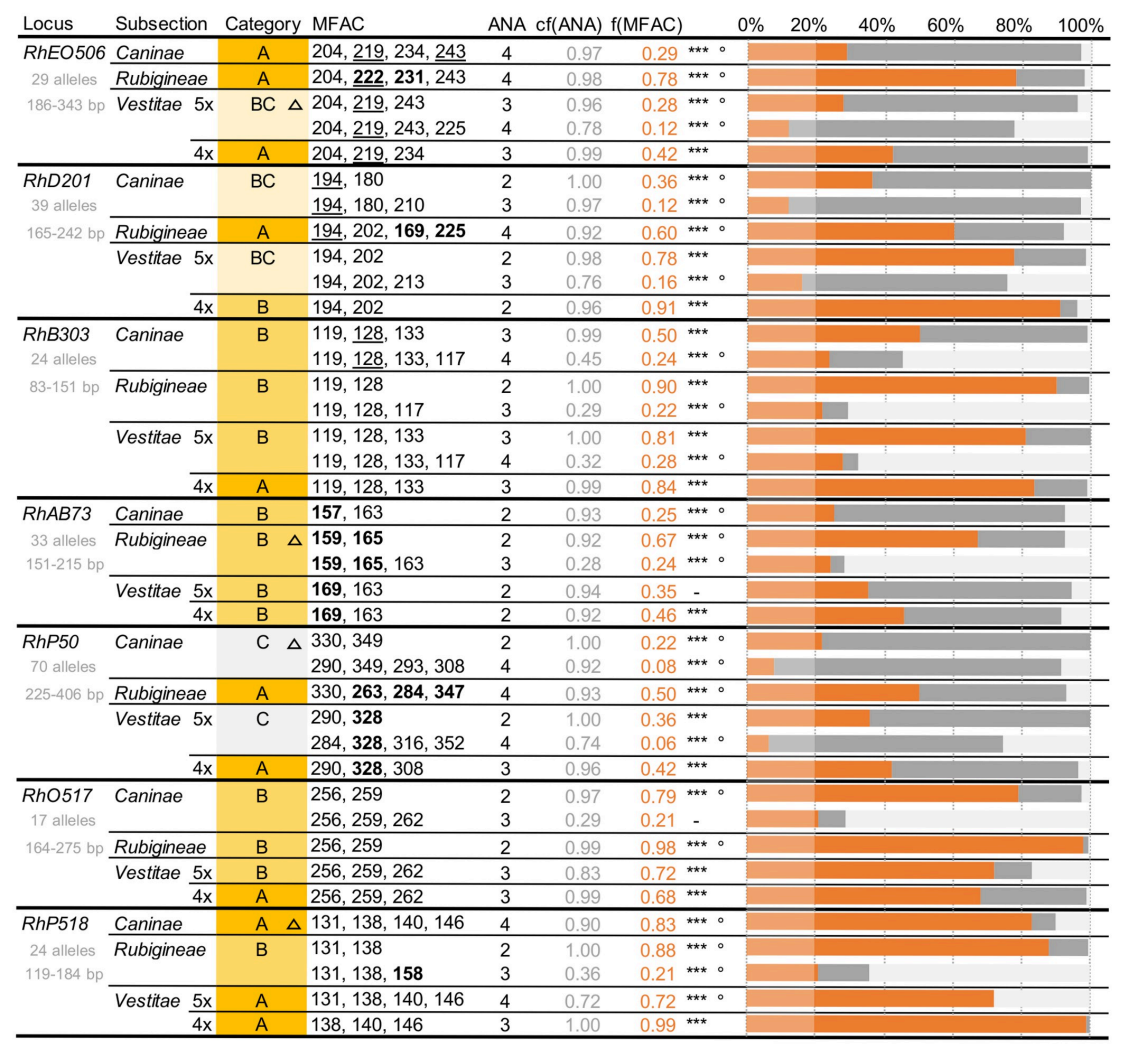
Most frequent allele combinations (MFACs) per locus and group. *Caninae* (5x) 958 samples; *Rubigineae* (5x) 315 samples; *Vestitae* (5x) 176 and (4x) 166 samples. Category–A: MFAC of ploidy-1 alleles; B: MFAC of less than ploidy-1 alleles; BC: MFAC hardly discernible; C: MFAC not discernible. Triangle: example for MFAC category shown in Fig 2; bold: MFAC / allele specific to subsection; underlined: allele proposed as bivalent in literature [28, 35]. ANA–apparent number of alleles in proposed MFAC; cf(ANA)–frequency of samples with at least ANA alleles in group (grey bar); f(MFAC)–frequency of MFAC in group (orange bar). ***: MFAC more frequent than random (p ≤ 0.001); °: MFAC more frequent in group than in entire dataset (p ≤ 0.01).

The most frequent allele combinations identified as likely candidates for co-inherited sets of microsatellite alleles resulting from an initial hybridization that gave rise to the respective *Caninae* subsection are displayed in Fig 5. As combinations of fewer alleles are generally more frequent than those of many, searching for a single, highly frequent candidate combination sometimes led to ambiguous results. We therefore sorted the locus-group combinations into different categories, which are illustrated by examples of the corresponding network plots in Fig 2 (complete plots in S2 File).

At loci in category A, we found a highly frequent combination of ploidy-minus-one alleles, which would likely correspond to the ancestral univalent allele combination and a nearly fixed bivalent allele. At loci in category B, we found a highly frequent combination of fewer than ploidy-minus-one alleles. This may correspond either to the ancestral univalent allele combination with more variable bivalent alleles, to the combination of univalent and bivalent alleles if one or more of the alleles on different sub-genomes have the same length, or to the presence of one or more null-alleles. For both categories A and B, we marked allele combinations that were specific to a certain subsection in bold; note that we did not distinguish between tetra- and pentaploid *Vestitae* here.

For loci in category BC, we had difficulties in determining the most frequent allele combinations. In two cases (5x *Vestitae* at loci *RhEO506* and *RhD201*), there were several nearly equally frequent allele combinations for the same number of alleles, which caused the frequency of each single combination to drop below 20%. In another case (*Caninae* at locus *RhD201*), the most frequent combination of four alleles was inconsistent with those for three or two alleles (S2 File). All three cases may have been caused by a high local mutation rate, by the presence of several nearly equally frequent bivalent alleles, by hidden genetic structure (although not detectable in the PCoA data for subsection *Caninae*, see Fig 4), or by multiple independent origins of the subsection.

For loci in category C, most samples had ploidy-minus-one alleles, but there was no single highly frequent four-allele combination. This scenario only occurred twice, and both times at the most allele-rich locus *RhP50*, suggesting a very high mutation rate at this locus is responsible. While the ancestral allele combination in pentaploid *Vestitae* at *RhP50* can still be inferred from the most frequent allele combination in tetraploid *Vestitae*, the ancestral allele combination in subsection *Caninae* at this locus remains unknown.

Of 28 locus-group combinations, 11 belonged to category A, 12 to category B, 3 to category BC and only 2 to category C. This appears to confirm the hypothesis that univalent alleles were inherited as a conserved set in the three subsections of *Rosa* section *Caninae* we studied. However, in at least 11 cases the coinherited sets contain more alleles than the number of univalent genomes, and must thus also include a highly conserved bivalent allele. Among the categories A and B, we found eight alleles or allele combinations that were specific to subsections. Five of these specific combinations characterized subsection *Rubigineae*, and only one was specific to the most sample-rich subsection *Caninae*. At locus *RhAB73* only, all subsections possessed a distinctive MFAC. At loci *Rh303*, *RhO517* and *RhP518*, the MFACs were identical between all subsections. While at least one allele in any subsection-specific MFAC was not found in any other MFAC, the alleles in subsection-specific MFACs were generally not exclusive to their subsection.

## Discussion

### Clonal richness does not generally differ between dogrose and non-dogrose species, nor does it correlate with geography

Our estimates on clonal richness within species were generally high (Fig 3), as would be expected if sampling vegetative offspring from the same plant was usually avoided. However, our results have to be taken with caution, since species assignment may be ambiguous, and they are based on allelic phenotypes. The dosage of alleles could not be determined, particularly in the polyploids. Consequently, both null and duplicate alleles were undetected, which may have caused physically different genotypes to be considered identical. Note that clonal richness is not linearly correlated with clonality [66], but if taken as such, it will usually lead to an underestimation of the true frequency of clonal reproduction.

In general, we did not find any geographic patterns of clonal richness between sites (Fig 1A), nor did we find any consistent differences in clonal richness between hemisexual and fully sexual roses (Fig 3). Instead, both hemisexuals in the *Caninae* subsection *Vestitae* and roses with regular meiosis had a higher proportion of identical MLGs than other hemisexual roses from the section *Caninae*. Comparing species with a different proportion of tetraploid samples within the subsection *Vestitae*, or the two sexual, tetraploid species *R*. *gallica* and *R*. *spinosissima*, this pattern is not correlated with ploidy.

Remarkably, we observed identical MLGs across huge distances of > 1 000 km. The largest distances and most frequent occurrence of a single MLG were in the subsection *Rubigineae*, although *R*. *canina* had the highest number of MLGs represented by more than one sample, and also the highest number of MLGs shared between sites > 50 km apart (Fig 1, S2 File). If not an artefact of the genetic marker system, human-mediated dispersal of wild roses, e.g. through cultivation in monastery gardens and subsequent dispersal from there [67], could have caused such patterns. Although the medicinal use, religious symbolism and widespread cultivation of wild rose species is well-known [68, 69], a dedicated study of human influence on their genetic structure has, to our knowledge, never been made. The results from our large-scale dataset indicate that this might be an interesting question to pursue.

### Genetic structure reflects *Rosa* sections and subsections, but not microspecies in the section *Caninae*

Major phylogenetic lineages, i.e. sections of the genus *Rosa*, are roughly mirrored by the patterns shown in the PCoA (Fig 4A). Samples of the sections *Rosa* and *Pimpinellifoliae* were arranged at one side of the plot along the first axis, while the sections *Gallicanae* and *Synstylae* had an intermediate position between the two former sections and the section *Caninae*. This separation into the rather early divergent taxa, the sections *Rosa* and *Pimpinellifoliae*, and the large *Synstylae* & allies clade (*Synstylae*, *Gallicanae*, *Caninae*) has also been revealed by various phylogenetic analyses based on plastid sequences [12, 39, 40, 70], whole genome approaches [9] and AFLP fingerprinting [21, 71], and it is reflected by specific rDNA patterns [38]. Remarkably, the tetraploid *R*. *gallica* was situated rather close to the section *Caninae*; both taxa are characterised by partially pinnatifid sepals. Based on rDNA clusters, *R*. *gallica* was hypothesized to belong to a potential parental lineage of the section *Caninae* [38].

Within the section *Caninae*, the subsections *Caninae* and *Rubigineae* were separated along the second axis of the PCoA analysis, whereas samples from the subsection *Vestitae* were split into two groups (Fig 4D), with one cluster partly overlapping with that of the subsection *Caninae* (Fig 4A). This finding is in accordance with AFLP analyses of the Generose samples (see Table 1; [21]), but in contrast to plastid phylogenies revealing the subsections *Rubigineae* and *Vestitae* as closely related and separated from the subsection *Caninae*, which rendered the entire section *Caninae* as polyphyletic [12, 40].

Within subsections, a meaningful substructure could only be detected within *Rubigineae* (Fig 4C), i.e. splitting *R*. *rubiginosa* agg. (roundish leaflet bases and glandular pedicels) from *R*. *inodora* agg. (cuneate leaflet bases and glandless pedicels), as has been previously shown by microsatellite data [13, 25]. The latter two publications suggested a further separation of hexaploid hybridogenic *R*. *micrantha (Rubigineae × Caninae)*, which is blurred in the present dataset. Yet, ploidy levels for this species were partially estimated from allele counts and not determined by flow cytometry for the present study (S1 File). Within the subsection *Vestitae*, samples split roughly by ploidy level and not by microspecies or aggregates, which is similar to findings for a subset of the present samples using AFLPs and microsatellites [14]. Thus, our large dataset does not support a sub-division into microspecies within subsections according to the L/D system. However, detailed analyses on more polymorphic sequence data might provide insights into ongoing hybridizations, and their outcomes might correlate with certain combinations of morphological characters that fit the description of microspecies. It remains debatable if such hybrids, which have originated several times independently [13], should be recognized as separate taxa at the species level.

Hybridogenic origins have also been hypothesized for some peculiar taxa within the section *Caninae*. Namely, the tetraploid *R*. *glauca* appeared to be intermediate between the sections *Caninae* and *Rosa* based on microsatellite data (Fig 4A) and AFLPs [21]. This is corroborated by the observation of canina meiosis [33] in this species, together with its nearly entire sepals, as typical for sect. *Rosa*. Similarly, *R*. *marginata* (subsection *Trachyphyllae*) is morphologically intermediate between the sections *Caninae* and *Gallicanae*, and it has been interpreted as a hybrid. However, even though admixture between *R*. *marginata* and *R*. *gallica* has been observed in Swiss populations [42], our data (Fig 4A) and AFLP data [21] do not support such a hypothesis.

### Due to canina meiosis, univalent alleles may be transmitted in lineage-specific sets

Overall, our statistical analysis of allele combinations showed the expected pattern of co-inherited alleles: In 23 out of 28 locus-group combinations, we found a clear candidate set for an ancestral allele combination in each of the three *Caninae* subsections (Fig 5). The patterns we found are not caused by the presence of repeat MLGs in our data, as samples identical at one locus need not be identical at all others. Excluding repeat MLGs from the dataset slightly changed the frequencies of individual allele combinations, but did not markedly alter the result (S2 File). Similarly, including samples with up to five different alleles per locus, i.e. potential hexaploid hybrids or pentaploid genotypes with heterozygous bivalent alleles, did not change the outcome (not shown). By running the same analyses at the microspecies level instead of the subsection level (not shown), we also tested whether the observed pattern could have been driven by individual sample-rich microspecies, but this was found not to be the case.

By contrast, the most frequent allele combinations we found were often highly similar and included few taxon-specific alleles or combinations (Fig 5). Across all seven studied loci, *Rubigineae* were more distinct from *Caninae* and *Vestitae*, which usually showed highly similar allele combinations. This seems contrary to the hypothesis that the subsections *Caninae* and *Rubiginae* originated form reciprocal crosses of the same ancestral lineages [36, 38], but it does concur with the results of a previous AFLP study [21], and with the results of a ddRAD study on a limited number of Swiss dogrose accessions [42]. The number of alleles involved in the potentially ancestral allele combinations at each locus roughly correlates to the locus’ overall allelic diversity, which ranged from three out of 17 alleles at *RhO517* to nine out of 70 alleles at *RhP50*. Based on these low numbers, a common origin of the three subsections from a restricted allele pool seems likely, be it by reciprocal crosses or by a different mechanism, e.g. spontaneous duplication of different ancestral genomes. Apart from that, we found no general patterns of similarity between the most frequent allele combinations, which indicates that their evolution is highly locus- and taxon-specific.

A major weakness of our data is that they do not allow us to distinguish between bivalent and univalent alleles. If, as has previously been suggested, the bivalent alleles are close to fixation in each respective subsection [28, 34], we would expect them to have been included in the most frequent allele combinations we determined. In the literature, few suggestions exist for the identity of bivalent alleles [28, 34, 35]: For example, allele 243 at locus *RhE506* has been suggested as a bivalent in *Caninae*, and it was indeed found in the MFAC, where it is the allele present in most samples from this subsection (Fig 5, S2 File). In *Rubigineae* and *Vestitae*, this allele is also found in the MFAC, and, as it should occur as a univalent there, it is indeed less prevalent across samples of the subsection *Vestitae* (but not in *Rubigineae*). By contrast, the same pattern is not true for the potential bivalent alleles 219 in *Vestitae* and 222 in *Rubigineae* at the same locus, which are also included in the respective MFACs, but they only rank second or even last in prevalence across samples when compared to the other MFAC alleles.

Although we have found a statistical approach that allows for the analysis of genetic diversity even in a highly irregular reproductive system such as canina meiosis, the results we achieved based on microsatellite fingerprint data are still far from conclusive. Some questions that still remain include whether the apparent “loss” of alleles from the MFAC at some loci is indicative of some extent of univalent recombination, of high allele homoplasy, or of mutations. Particularly in the latter case, why would plants with heterozygous bivalent alleles be rare? Does recurrent recombination of the bivalent alleles render them less diverse than their univalent counterparts (comparable to a “Meselson effect”, [72])? What are the actual differences between clonal and sexual genome evolution in hemisexual roses–if not relative to diversity as such, does the speed of evolution differ (compare e.g. [73])? While microsatellites were an economical and sufficiently reproducible method to generate the extensive, Europe-wide dataset on which this study is based, their resolution is not high enough to efficiently tackle these questions. Although hemisexual, polyploid inheritance with canina meiosis may be too idiosyncratic to allow for a direct transferal of our analysis method to other systems, it can be re-used once more genetic data becomes available, and it may serve as inspiration for ways to untangle the evolution of other aneuploid and irregular inheritance systems.

## Conclusion

Our Europe-wide analysis of genetic diversity in wild roses revealed several results that go beyond those of previous separate studies on the three source datasets. The analyses of the taxonomic and spatial distribution of MLGs showed that clonal propagation, despite the apparently small dispersal range of root suckers or apomictic rose seeds, reached much further than suspected, which is likely due to human intervention. However, it also suggested a considerable rate of “misidentification” of samples with identical MLGs from the same site as belonging to different morpho-species. This tallies with the result that subsections, rather than morphology-based microspecies, appear to be genetically distinct evolutionary units within the *Rosa* section *Caninae*, although morphologically stable inter-sectional hybrids may also occur. The occurrence of highly frequent sets of apparently co-inherited alleles, which in some cases even are specific to different subsections, suggests a common origin for each subsection and further lends credence to their status as distinct evolutionary lineages. We found that the reliability of our results is somewhat compromised by the lack of allele dosage information and the potential homoplasy of same-length alleles. Ideally, we would also have wished for our samples to be more geographically even and representatively distributed, which is still hard to achieve at this spatial scale. However, our present results may serve as a basis upon which future studies, involving higher-resolution genetic markers or even phased genomic data [74], can build.

## Supporting information

S1 File Dataset and metadata. (XLSX)

S2 File Additional figures. (PDF)

S3 File Laboratory methods. (PDF)

## References

[1] Grant V. Plant speciation. New York, London: Columbia University Press; 1971. 369–77 p.

[2] Hojsgaard D, Hörandl E. The rise of apomixis in natural plant populations. Frontiers in Plant Science. 2019;10. doi: 10.3389/fpls.2019.00358 WOS:000463028200001. 31001296 PMC6454013

[3] Soltis PS, Soltis DE. The role of hybridization in plant speciation. Annual Review of Plant Biology. 2009;60:561–88. doi: 10.1146/annurev.arplant.043008.092039 WOS:000268071800024. 19575590

[4] Carman JG. Do duplicate genes cause apomixis? In: Hörandl E, Grossniklaus U, van Dijk PJ, Sharbel TF, editors. Apomixis–Evolution, mechanisms and perspectives. Vienna: International Association of Plant Taxonomy; 2007.

[5] Mayrose I, Zhan SH, Rothfels CJ, Magnuson-Ford K, Barker MS, Rieseberg LH, et al. Recently formed polyploid plants diversify at lower rates. Science. 2011;333(6047):1257–. WOS:000294406400044. doi: 10.1126/science.1207205 21852456

[6] Pelé A, Rousseau-Gueutin M, Chevre AM. Speciation success of polyploid plants closely relates to the regulation of meiotic recombination. Frontiers in Plant Science. 2018;9. WOS:000436569700001. 10.3389/fpls.2018.00907 PMC6031745 30002669

[7] Soltis DE, Visger CJ, Soltis PS. The polyploidy revolution then…and now: Stebbins revisited. American Journal of Botany. 2014;101(17):1057–78. doi: 10.3732/ajb.1400178 25049267

[8] Dressler S, Gregor T, Hellwig FH, Korsch H, Wesche K, Wesenberg J, et al. Comprehensive and reliable–a new virtual herbarium of critical plant taxa in Germany. Plant Systematics and Evolution. 2017;303(8):1109–13.

[9] Debray K, Le Paslier MC, Berard A, Thouroude T, Michel G, Marie-Magdelaine J, et al. Unveiling the patterns of reticulated evolutionary processes with phylogenomics: hybridization and polyploidy in the genus *Rosa*. Systemic Biology. 2021. doi: 10.1093/sysbio/syab064 WOS:000764757000001. 34329460

[10] Joly S, Starr JR, Lewis WH, Bruneau A. Polyploid and hybrid evolution in roses east of the Rocky Mountains. American Journal of Botany. 2006;93(3):412–25. doi: 10.3732/ajb.93.3.412 21646201

[11] Ritz CM, Schmuths H, Wissemann V. Evolution by reticulation: European dogroses originated by multiple hybridization across the genus *Rosa*. Journal of Heredity. 2005;96:4–14. 15618309 10.1093/jhered/esi011

[12] Fougere-Danezan M, Joly S, Bruneau A, Gao XF, Zhang LB. Phylogeny and biogeography of wild roses with specific attention to polyploids. Annals of Botany. 2015;115:275–91. doi: 10.1093/aob/mcu245 25550144 PMC4551085

[13] Herklotz V, Ritz CM. Multiple and asymmetric origin of polyploid dogrose hybrids (Rosa L. sect. Caninae (DC.) Ser.) involving unreduced gametes. Annals of Botany. 2017;120:209–20. doi: 10.1093/aob/mcw217 28028016 PMC5737388

[14] Kellner A, Ritz CM, Wissemann V. Low genetic and morphological differentiation in the European species complex of *Rosa sherardii*, *R*. *mollis* and *R*. *villosa* (*Rosa* section *Caninae* subsection *Vestitae*). Botanical Journal of the Linnean Society. 2014;174(2):240–56. doi: 10.1111/boj.12124 WOS:000329686000006.

[15] Klásteršká I. Cytologyand some chromosome numbers of Czechoslovak roses I. Folia Geobotanica et Phytotaxonomica. 1969;4(2):175–89.

[16] Klásteršká I. Cytology and chromosome numbers of Czechoslovak roses II. Folia Geobotanica et Phytotaxonomica. 1969;4(2):175–89.

[17] Klásteršká I, Natarajan AT. Cytological studies of the genus *Rosa* with special reference to section *Caninae*. Hereditas. 1974;76:97–108. 4136006 10.1111/j.1601-5223.1974.tb01181.x

[18] Małecka J, Popek R. Karyological studies in the Polish representatives of the genus Rosa L. I. Acta Biologica Cracoviensia Series Botanica. 1982;14:79–90.

[19] Małecka J, Popek R. Karyological studies in the Polish representatives of the genus Rosa L. II. Acta Biologica Cracoviensia Series Botanica. 1984;16:43–54.

[20] Pachl Š. Variablita botanických druhů rodu *Rosa* L., a možosti jejich využití v krajinářské tvorbê. Nitra, Slovakia: Slovak University of Agriculture; 2011.

[21] De Riek J, De Cock K, Smulders MJM, Nybom H. AFLP-based population structure analysis as a means to validate the complex taxonomy of dogroses (*Rosa* section *Caninae*). Molecular Phylogenetics and Evolution. 2013;67(3):547–59. 10.1016/j.ympev.2013.02.024. WOS:000317996100001. 23499615

[22] Christ H. Die Rosen der Schweiz. Basel: Verlag H. Georg; 1873.

[23] Dingler H. Versuch einer Erklärung gewisser Erscheinungen in der Ausbildung und Verbreitung der wilden Rosen. Mitteilungen des Naturwissenschaftlichen Vereins Aschaffenburg. 1907;6:1–38.

[24] Reichert H. Die zwei Wuchstypen bei Rosen der Sektion Caninae und ein Vorschlag für eine Kurzbezeichnung derselben. Acta Rhodologica. 1998;1:29–35.

[25] Herklotz V, Mieder N, Ritz CM. Cytological, genetic and morphological variation in mixed stands of dogroses (*Rosa* L. sect. *Caninae* (DC.) Ser.) in Germany with a focus on the hybridogenic *R*. *micrantha* Sm. Botanical Journal of the Linnean Society. 2017;184:254–71.

[26] Schanzer IA, Kutlunina NA. Interspecific hybridization in wild roses (*Rosa* L. sect. *Caninae* DC.). Biology Bulletin. 2010;37(5):480–8. WOS:000282182400007. 21077366

[27] Schanzer IA, Vagina AV. ISSR (Inter Simple Sequence Repeat) markers reveal natural intersectional hybridization in wild roses [*Rosa* L., sect. *Caninae* (DC.) Ser. and sect. *Cinnamomeae* (DC.) Ser.]. Wulfenia. 2007;14:1–14. ISI:000256305700001.

[28] Nybom H, Esselink GD, Werlemark G, Vosman B. Microsatellite DNA marker inheritance indicates preferential pairing between two highly homologous genomes in polyploid and hemisexual dog-roses, *Rosa* L. sect. *Caninae* DC. Heredity. 2004;92(3):139–50. 10.1038/sj.hdy.6800332. ISI:000189168600004. 14981531

[29] Werlemark G, Nybom H, Olsson A, Uggla M. Variation and inheritance in hemisexual dogroses, *Rosa* section *Caninae*. Biotechnol & Biotechnol Eq. 2000;14:28–31.

[30] Blackburn KB. Chromosomes and classification in the genus *Rosa*. American Naturalist. 1925;59:200–4.

[31] Blackburn KB, Harrison JHW. The status of the British rose forms as determined by their cytological behaviour. Annals of Botany. 1921;35(138):159–88.

[32] Täckholm G. On the cytology of the genus *Rosa*. A preliminary note. Svensk Botanisk Tidskrift. 1920;14(2–3):300–11.

[33] Täckholm G. Zytologische Studien über die Gattung *Rosa*. Acta Horti Bergiani. 1922;7:97–381.

[34] Nybom H, Esselink GD, Werlemark G, Leus L, Vosman B. Unique genomic configuration revealed by microsatellite DNA in polyploid dogroses, *Rosa* sect. *Caninae*. Journal of Evolutionary Biology. 2006;19(2):635–48. doi: 10.1111/j.1420-9101.2005.01010.x 16599938

[35] Ritz CM, Wissemann V. Microsatellite analyses of artificial and spontaneous dogrose hybrids reveal the hybridogenic origin of *Rosa micrantha* by the contribution of unreduced gametes. Journal of Heredity. 2011;102(2):217–27. 21220743 10.1093/jhered/esq124

[36] Herklotz V, Kovařík A, Lunerová L, Lippitsch S, Groth M, Ritz CM. The fate of ribosomal RNA genes in spontaneous polyploid dogrose hybrids (*Rosa* L. sect. *Caninae* (DC.) Ser.) exhibiting non-symmetrical meiosis. Plant Journal. 2018;94(1):77–90. 10.1111/tpj.13843 29385286

[37] Lunerová L, Herklotz V, Laudien M, Vozárová R, Groth M, Kovařík A, et al. Asymmetrical canina meiosis is accompanied by the expansion of a pericentromeric satellite in non-recombining univalent chromosomes. Annals of Botany. 2020;125(7):1025–38. 32095807 10.1093/aob/mcaa028 PMC7262465

[38] Vozárová R, Herklotz V, Kovařík A, Tynkevich YO, Volkov RA, CM R, et al. Ancient origin of two 5S rDNA families dominating in the genus *Rosa* and their behaviour in the canina-type meiosis. Frontiers in Plant Science. 2021;12:Article 643548. doi: 10.3389/fpls.2021.643548 33763100 PMC7984461

[39] Bruneau A, Starr JR, Joly S. Phylogenetic relationships in the genus *Rosa*: new evidence from chloroplast DNA sequences and an appraisal of current knowledge. Systematic Botany. 2007;32(2):366–78. doi: 10.1600/036364407781179653

[40] Wissemann V, Ritz CM. The genus *Rosa* (Rosoideae, Rosaceae) revisited: molecular analysis of nrITS-1 and *atp*B-*rbc*L intergenic spacer (IGS) versus conventional taxonomy. Botanical Journal of the Linnean Society. 2005;147:275–90. doi: 10.1111/j.1095-8339.2005.00368.x

[41] De Cock K, Vander Mijnsbrugge K, Breyne P, Nybom H, Smulders MJM, Van Slycken J, et al. The diversity of autochthonous roses in Flanders (Belgium) in view of the European GENEROSE reference framework. Acta Horticulturae (Wageningen). 2007;760(621–628).

[42] Ballmer D. Dogrose evolution and its implications for conservation [MSc-Thesis]: University Zuerich (UZH); 2018.

[43] Herklotz V, Ritz CM. Spontane Hybridisierung von Hundsrosen (Rosa L. sect. Caninae (DC). Ser.) an einem natürlichen Vorkommen in der Oberlausitz (Sachsen, Deutschland). Peckiana. 2014;9:1–12.

[44] Kellner A, Ritz CM, Wissemann V. Hybridization with invasive *Rosa rugosa* threatens the genetic integrity of native *Rosa mollis*. Botanical Journal of the Linnean Society. 2012;170(3):472–84. doi: 10.1111/j.1095-8339.2012.01298.x WOS:000310079500008.

[45] Van Huylenbroeck J, Smulders MJM, Debener T, Nybom H, Gudin S, Cox P, et al. GENEROSE: Genetic evaluation of European rose resources for conservation and horticultural use. Acta Horticulturae (ISHS). 2005;690:119–24. 10.17660/ActaHortic.2005.690.17

[46] Henker H. *Rosa*. In: Conert HJ, Jäger EJ, Kadereit JW, Schultze-Motel W, Wagenitz G, Weber HE, editors. Gustav Hegi–Illustrierte Flora von Mitteleuropa. IV/2C. Berlin: Parey; 2000. p. 1–108.

[47] Esselink GD, Smulders MJM, Vosman B. Identification of cut rose (*Rosa hybrida*) and rootstock varieties using robust sequence tagged microsatellite site markers. Theoretical and Applied Genetics. 2003;106:277–86. 10.1007/s00122-002-1122-y 12582853

[48] Zhang J, Esselink GD, Che D, Fougere-Danezan M, Arens P, Smulders MJM. The diploid origins of allopolyploid rose species studied using single nucleotide polymorphism haplotypes flanking a microsatellite repeat. Journal of Horticultural Science & Biotechnology. 2013;88(1):85–92. doi: 10.1080/14620316.2013.11512940 WOS:000322941500011.

[49] Roberts AV, Gladis T, Brumme H. DNA amounts of roses (*Rosa* L.) and their use in attributing ploidy levels. Plant Cell Reports. 2009;28(1):61–71. doi: 10.1007/s00299-008-0615-9 WOS:000261655900007. 18839177

[50] Yokoya K, Roberts V, Mottley J, Lewis R, Brandham PE. Nuclear DNA amounts in roses. Annals of Botany. 2000;85:557–61.

[51] Dorken ME, Eckert CG. Severely reduced sexual reproduction in northern populations of a clonal plant, *Decodon verticillatus* (Lythraceae). Journal of Ecology. 2001;89(3):339–50. doi: 10.1046/j.1365-2745.2001.00558.x WOS:000169689900002.

[52] Python 3.7.0 [Internet]. 2018 [cited 1.1.2023]. Available from: https://www.python.org/.

[53] Pandas 0.25.1 [Internet]. 2019 [cited 1.1.2023]. Available from: https://pandas.pydata.org/pandas-docs/version/0.25.1/index.html.

[54] QGIS Development Team. Quantum GIS (QGIS Geographic Information System. Open Source Geospatial Foundation Project 2018. Available from: http://download.osgeo.org/qgis/win64/.

[55] R-Core-Team. R: A language and environment for statistical computing. Vienna, Austria: R Foundation for Statistical Computing; 2015.

[56] Wickham H. ggplot2: elegant graphics for data analysis. New York: Springer; 2016.

[57] Paradis E, Schliep K. ape 5.0: an environment for modern phylogenetics and evolutionary analyses in R. Bioinformatics. 2019;35(3):526–8. doi: 10.1093/bioinformatics/bty633 WOS:000459315700023. 30016406

[58] Bruvo R, Michiels NK, D’Souza TG, Schulenburg H. A simple method for the calculation of microsatellite genotype distances irrespective of ploidy level. Molecular Ecology. 2004;13(7):2101–6. doi: 10.1111/j.1365-294X.2004.02209.x 15189230

[59] Clark LV, Jaseniuk M. POLYSAT: an R package for polyploid microsatellite analysis. Molecular Ecology Resources. 2011;11(3):562–6. doi: 10.1111/j.1755-0998.2011.02985.x 21481215

[60] Anmarkrud JA, Kleven O, Bachmann L, Lifjeld JT. Microsatellite evolution: mutations, sequence variation, and homoplasy in the hypervariable avian microsatellite locus HrU10. BMC Evolutionary Biology. 2008;8. doi: 10.1186/1471-2148-8-138 WOS:000256402200003. 18471288 PMC2396632

[61] Brohede J, Primmer CR, Moller A, Ellegren H. Heterogeneity in the rate and pattern of germline mutation at individual microsatellite loci. Nucleic Acids Research. 2002;30(9):1997–2003. doi: 10.1093/nar/30.9.1997 WOS:000175591100017. 11972338 PMC113841

[62] Kosman E, Jokela J. Dissimilarity of individual microsatellite profiles under different mutation models: Empirical approach. Ecology and Evolution. 2019;9(7):4038–54. doi: 10.1002/ece3.5032 WOS:000465089500030. 31015986 PMC6467862

[63] Meirmans PG, Liu SL, van Tienderen PH. The analysis of polyploid genetic data. Journal of Heredity. 2018;109(3):283–96. doi: 10.1093/jhered/esy006 WOS:000427844900007. 29385510

[64] Kamvar ZN, Brooks JC, Grünwald NJ. Novel R tools for analysis of genome-wide population genetic data with emphasis on clonality. Frontiers in Genetics. 2015;6(208). doi: 10.3389/fgene.2015.00208 26113860 PMC4462096

[65] Kamvar ZN, Tabima JF, Grünwald NJ. Poppr: an R package for genetic analysis of populations with clonal, partially clonal, and/or sexual reproduction. PeerJ. 2014;2(e281). doi: 10.7717/peerj.281 24688859 PMC3961149

[66] Stoeckel S, Porro B, Arnaud-Haond S. The discernible and hidden effects of clonality on the genotypic and genetic states of populations: Improving our estimation of clonal rates. Molecular Ecology Resources. 2021;21(4):1068–84. doi: 10.1111/1755-0998.13316 WOS:000610842200001. 33386695

[67] Åsen P. Medieval monastery gardens in Iceland and Norway. Religions. 2021;12:317. 10.3390/rel12050317.

[68] Rosen Krüssmann G., Rosen, Rosen: unser Wissen über die Rose. Berlin, Hamburg: Paul Parey; 1986.

[69] Touw M. Roses in the Middle-Ages. Economic Botany. 1982;36(1):71–83. doi: 10.1007/bf02858701 WOS:A1982ND19500004.

[70] Zhang C, Li S-Q, Xie H-H, Liu J-Q, Gao X-F. Comparative plastid genome analyses of *Rosa*: Insights into the phylogeny and gene divergence. Tree Genetics & Genomes. 2022;18:20. doi: 10.1007/s11295-022-01549-8

[71] Koopman WJM, Wissemann V, De Cock K, Van Huylenbroeck J, De Riek J, Sabatino GJH, et al. AFLP markers as a tool to reconstruct complex relationships: A case study in *Rosa* (Rosaceae). American Journal of Botany. 2008;95(3):353–66. doi: 10.3732/ajb.95.3.353 ISI:000253999600011. 21632360

[72] Birky CW. Heterozygosity, heteromorphy, and phylogenetic trees in asexual eukaryotes. Genetics. 1996;144(1):427–37. WOS:A1996VE25200039. doi: 10.1093/genetics/144.1.427 8878706 PMC1207515

[73] Reichel K, Masson JP, Malrieu F, Arnaud-Haond S, Stoeckel S. Rare sex or out of reach equilibrium? The dynamics of FIS in partially clonal organisms. BMC Genetics. 2016;17. doi: 10.1186/s12863-016-0388-z WOS:000377578300002. 27286682 PMC4902967

[74] Smulders MJM, Arens P, Bourke PM, Debener T, Linde M, De Riek J, et al. In the name of the rose: a roadmap for rose research in the genome era. Horticulture Research. 2019;6:65. doi: 10.1038/s41438-019-0156-0 31069087 PMC6499834

