# Supplementary material for: Untangling the hedge: Genetic diversity in clonally and sexually transmitted genomes of European wild roses, Rosa L
Source: PLoS One. 2023 Oct 5;18(10):e0292634. doi: 10.1371/journal.pone.0292634 (PMC10553836; doi:10.1371/journal.pone.0292634)
Supplement: S2 File — (PDF) [file pone.0292634.s002.pdf]

Reichel et al. 2023 – Genetic diversity in European roses

Supplementary Figures

Content

Figure S2-1 – MLGs shared between *Rosa* microspecies

Figure S2-2 – Sites with identical MLGs > 50 km apart

Figure S2-3 – Shannon index and evenness of MLGs

Figure S2-4 – Allele prevalence and apparent number of alleles per locus

Figure S2-5 – Network plots for subsection *Caninae*

Figure S2-6 – Network plots for subsection *Rubigineae*

Figure S2-7 – Network plots for subsection *Vestitae*: presumably pentaploid samples

Figure S2-8 – Network plots for subsection *Vestitae*: presumably tetraploid samples

Figures S2-9 to S2-12 – same as S2-5 to S2-8, but with one sample per MLG

Figure S2-13 – PCoA with geographic information

Section	Subsection	Species	R. caesia	R. canina	R. corymbifera	R. dumalis	R. stylosa	R. subcanina	R. subcollina	R. agrestis	R. elliptica	R. gremlii	R. inodora	R. micrantha	R. rubiginosa	R. glauca	R. balsamica	R. marginata	R. mollis	R. pseudoscabriuscula	R. sherardii	R. tomentosa	R. villosa	R. gallica	R. spinosissima	R. majalis	R. pendulina	R. arvensis
Section			Caninae	Caninae	Caninae	Caninae	Caninae	Caninae	Caninae	Caninae	Caninae	Caninae	Caninae	Caninae	Caninae	Caninae	Caninae	Caninae	Caninae	Caninae	Caninae	Caninae	Caninae	Gallicanae	Pimpinellifoliae	Rosa	Rosa	Synstylae
	Subsection		Caninae	Caninae	Caninae	Caninae	Caninae	Caninae	Caninae	Rubigineae	Rubigineae	Rubigineae	Rubigineae	Rubigineae	Rubigineae	Rubrifoliae	Tomentellae	Trachyphyllae	Vestitae	Vestitae	Vestitae	Vestitae	Vestitae					
Caninae	Caninae	R. caesia	1																	1		1						
Caninae	Caninae	R. canina		97	2																							
Caninae	Caninae	R. corymbifera		3	20												2	1										
Caninae	Caninae	R. dumalis				4		1	1																			
Caninae	Caninae	R. stylosa					1																					
Caninae	Caninae	R. subcanina		6				0	1																			
Caninae	Caninae	R. subcollina							3																			
Caninae	Rubigineae	R. agrestis								5				1														
Caninae	Rubigineae	R. elliptica									0		1															
Caninae	Rubigineae	R. gremlii										15		1	3													
Caninae	Rubigineae	R. inodora								1		1	4															
Caninae	Rubigineae	R. micrantha								1		6		10														
Caninae	Rubigineae	R. rubiginosa								1		5		1	7													
Caninae	Rubrifoliae	R. glauca														1												
Caninae	Tomentellae	R. balsamica										1			1		8											
Caninae	Trachyphyllae	R. marginata																2										
Caninae	Vestitae	R. mollis																	9									
Caninae	Vestitae	R. pseudoscabriuscula																		1		1						
Caninae	Vestitae	R. sherardii																			11		4					
Caninae	Vestitae	R. tomentosa															1					10						
Caninae	Vestitae	R. villosa																			1		27					
Gallicanae		R. gallica																						14				
Pimpinellifoliae		R. spinosissima																							33			
Rosa		R. majalis																								4		
Rosa		R. pendulina																									2	
Synstylae		R. arvensis																										6

different site

same site

Figure S2-1. MLGs shared between *Rosa* microspecies. Diagonal – shared MLGs within each species, at same or different sampling site. Above diagonal (black) – shared MLGs found at different sites. Below diagonal (red) – shared MLGs found at the same site. MLGs shared among three species were counted as in three species pairs (i.e. twice per species).

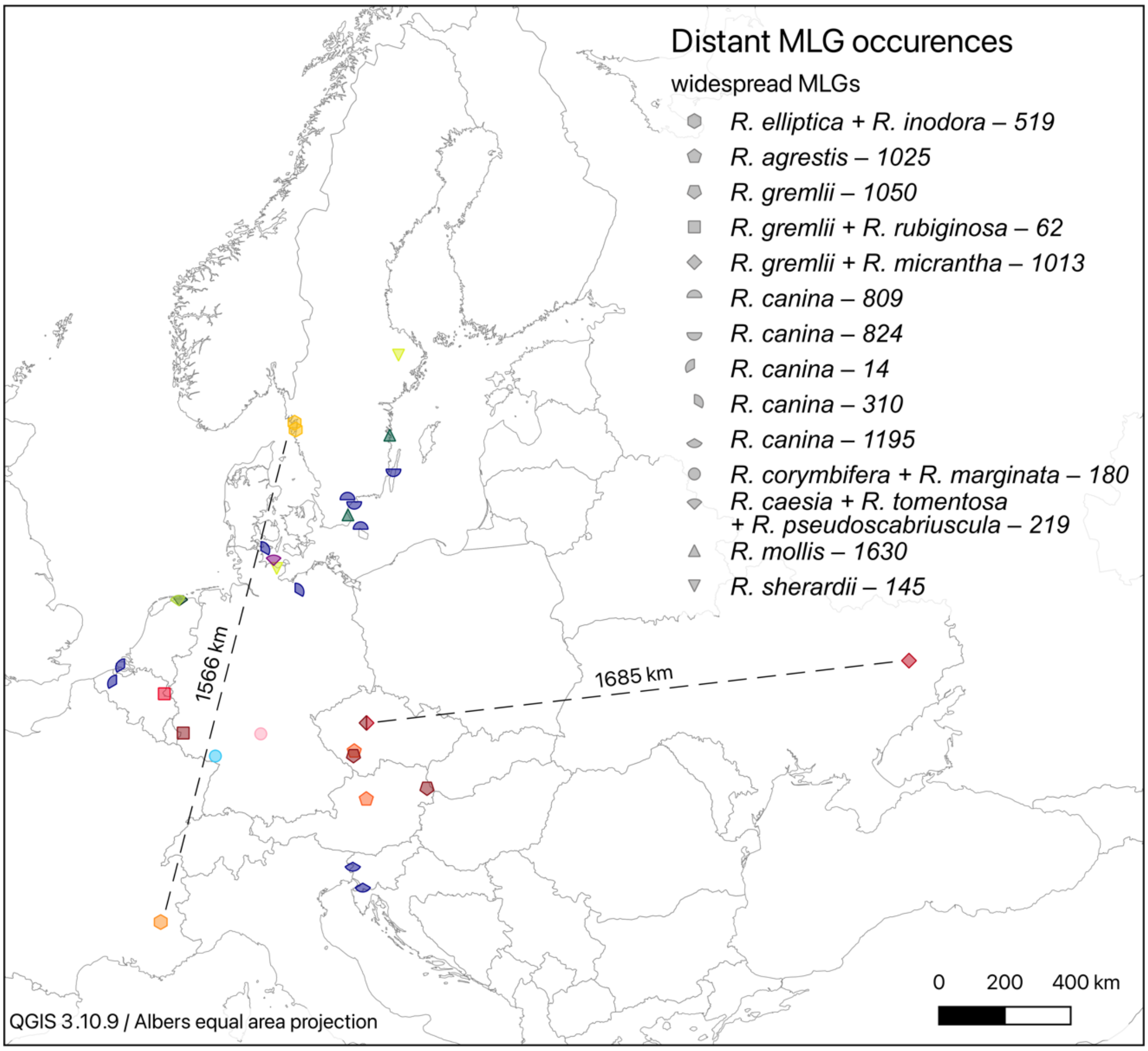

Figure S2-2. Sites with identical MLGs > 50 km apart. Dashed lines denote the largest distances between pairs, colors indicate species (see Fig 3 and 4 in the main text) and symbols distinct MLGs. For MLG 519 (3 sites), only the single sample from France had been assigned to *R. elliptica*. For MLG 1013, both samples from Ukraine had been assigned to *R. micrantha,* just like two of the five samples from the Czech Republic. Country outlines from Natural Earth.

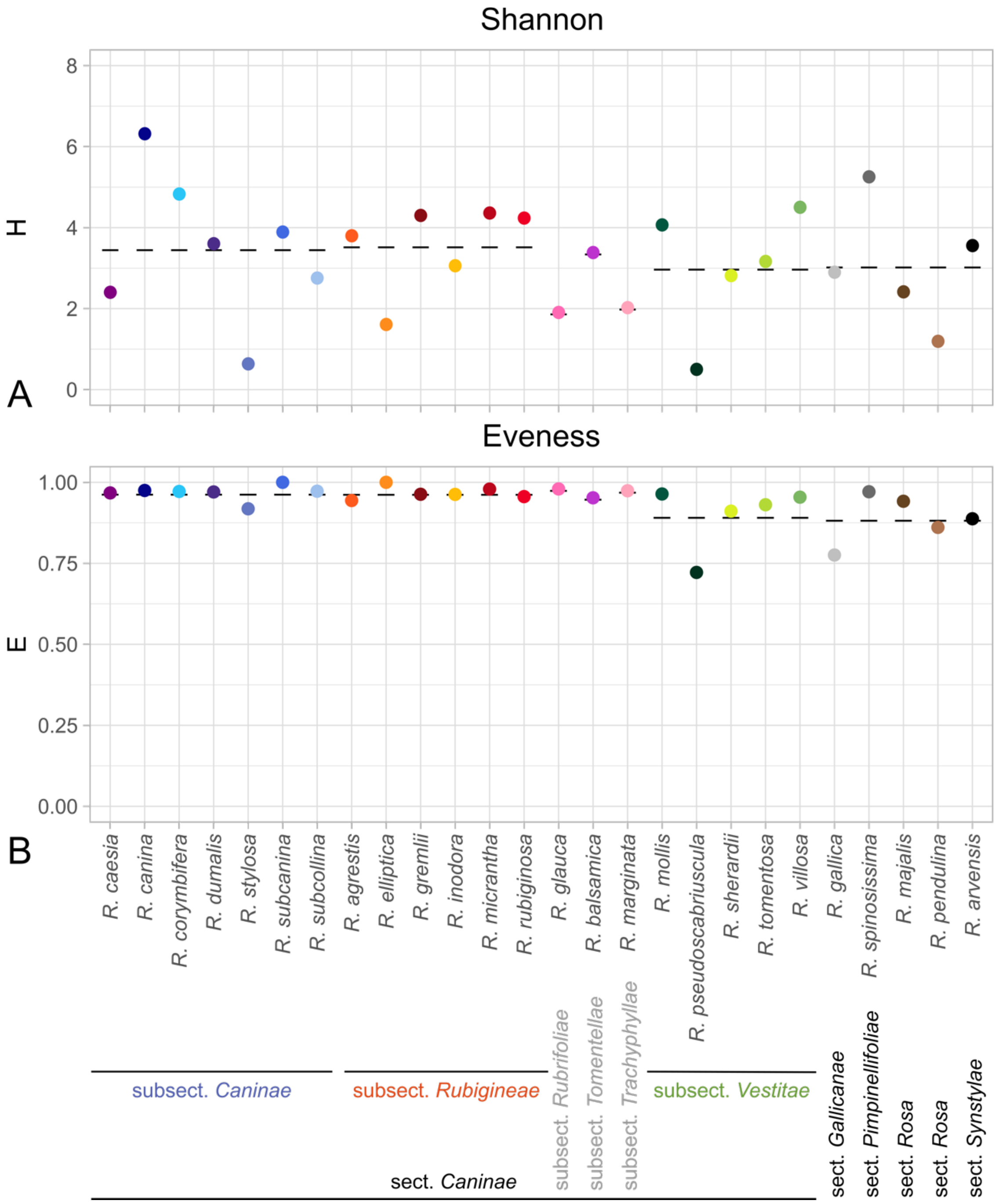

Figure S2-3 Shannon index and Evenness of MLGs. (A) Shannon index, (B) Evenness, calculated for each microspecies, distinguishing between identical MLGs from different sites and/or species.

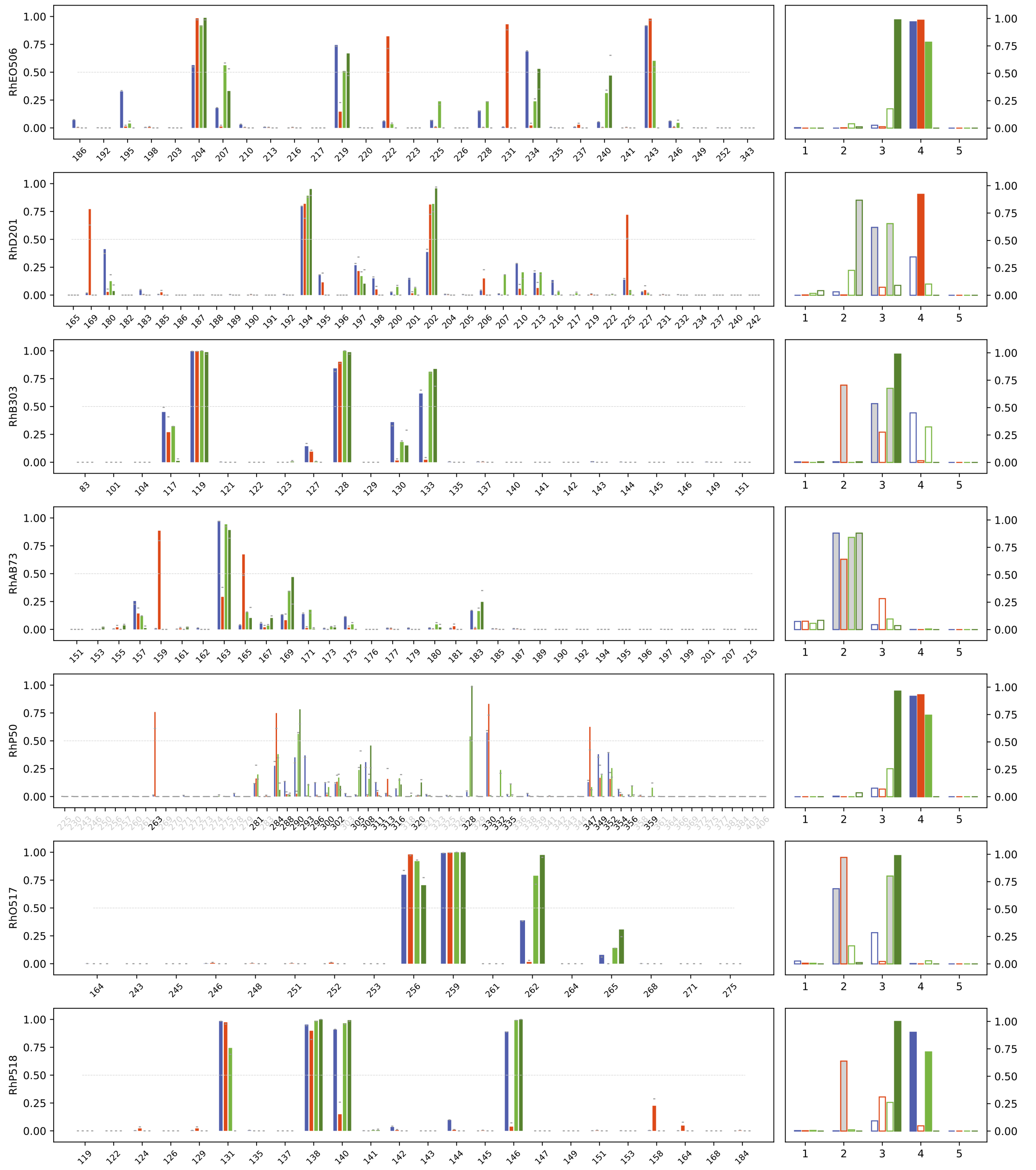

Figure S2-4. Allele prevalence and apparent number of alleles per locus. Left column: Allele prevalence, i.e. proportions of samples containing the respective allele for subsections *Caninae* (blue), *Rubigineae* (red), 5x (light green) and 4x *Vestitae* (dark green) at each of the seven microsatellite loci. Numbers on the x-axis denote allele lengths, for RhP50 partially greyed out to improve readability. Right column: Histograms of apparent numbers of alleles, i.e. proportions of samples containing 1 to 5 different alleles per locus in subsections *Caninae, Rubigineae*, 4x *Vestitae* and 5x *Vestitae* at each of the seven microsatellite loci. Median per subsection marked by filled bar, filled in grey if median below expectation for the respective ploidy level. Numbers on the x-axis denote ploidy levels.

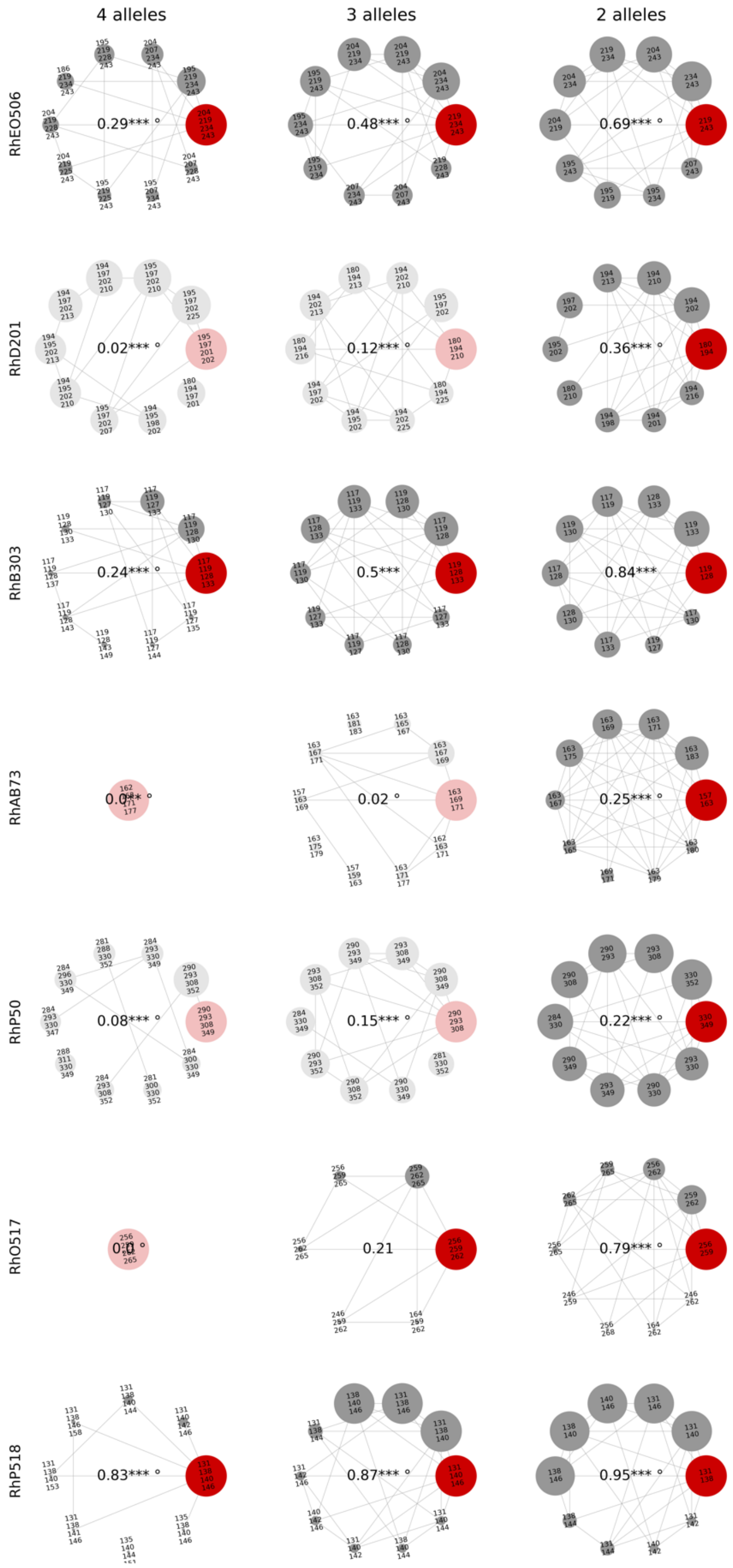

Figure S2-5. Network plots for subsection *Caninae*. Allele combinations differing by only one allele are connected by lines. Circle diameter corresponds to the frequency of the respective allele combination, relative to the most frequent combination (MFAC, in red, maximum diameter). The frequency of the MFAC is displayed in the center of the network, marked by stars if significantly higher than random (* $p \leq 0.05$, ** $p \leq 0.01$, *** $p \leq 0.001$), and by a degree sign if significantly higher within the respective subsection (° $p \leq 0.001$).

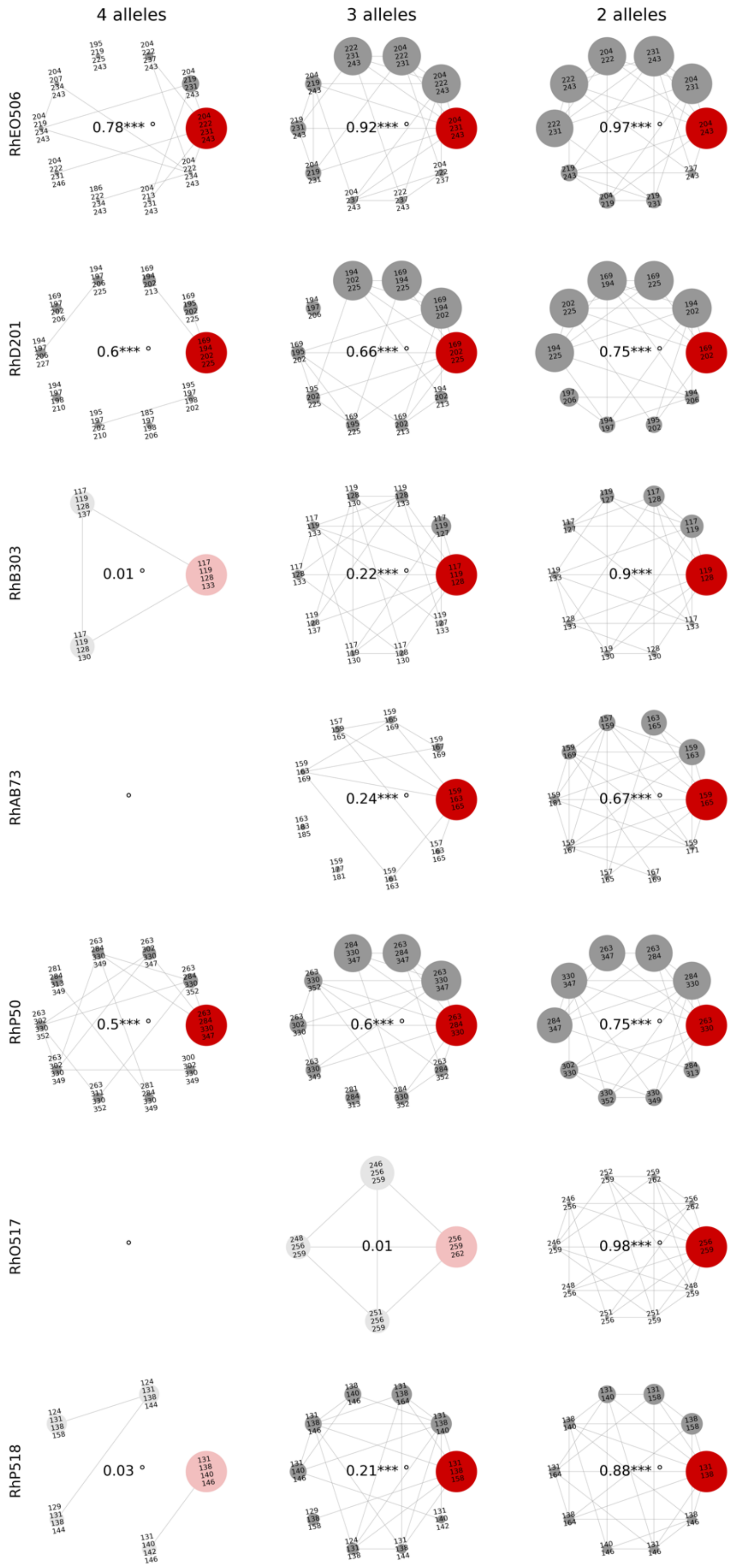

Figure S2-6. Network plots for subsection *Rubigineae*. Allele combinations differing by only one allele are connected by lines. Circle diameter corresponds to the frequency of the respective allele combination, relative to the most frequent combination (MFAC, in red, maximum diameter). The frequency of the MFAC is displayed in the center of the network, marked by stars if significantly higher than random (* p ≤ 0.05, ** p ≤ 0.01, *** p ≤ 0.001), and by a degree sign if significantly higher within the respective subsection (° p ≤ 0.001).

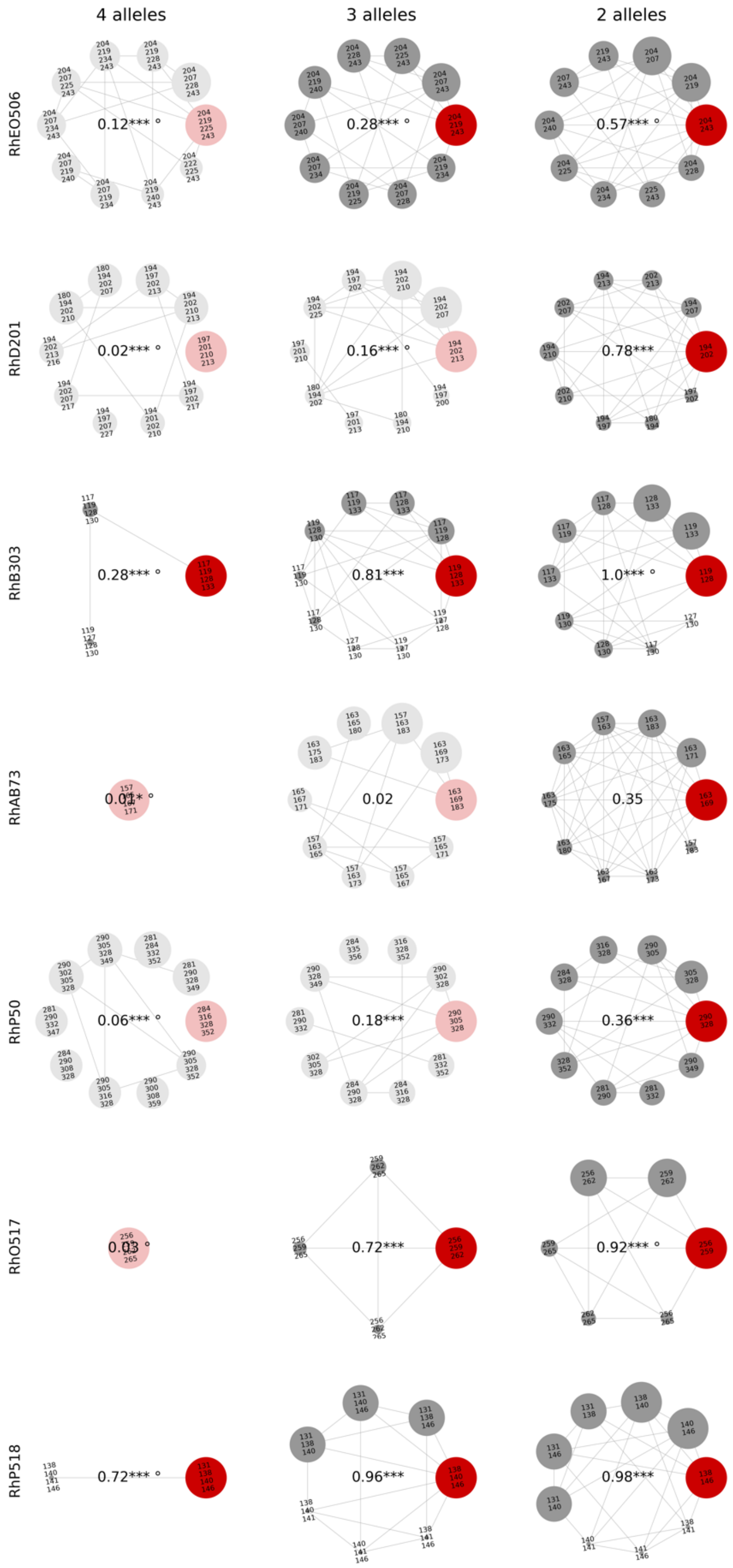

Figure S2-7. Network plots for subsection *Vestitae*: presumably pentaploid samples. Allele combinations differing by only one allele are connected by lines. Circle diameter corresponds to the frequency of the respective allele combination, relative to the most frequent combination (MFAC, in red, maximum diameter). The frequency of the MFAC is displayed in the center of the network, marked by stars if significantly higher than random (* $p \leq 0.05$, ** $p \leq 0.01$, *** $p \leq 0.001$), and by a degree sign if significantly higher within the respective subsection (° $p \leq 0.001$).

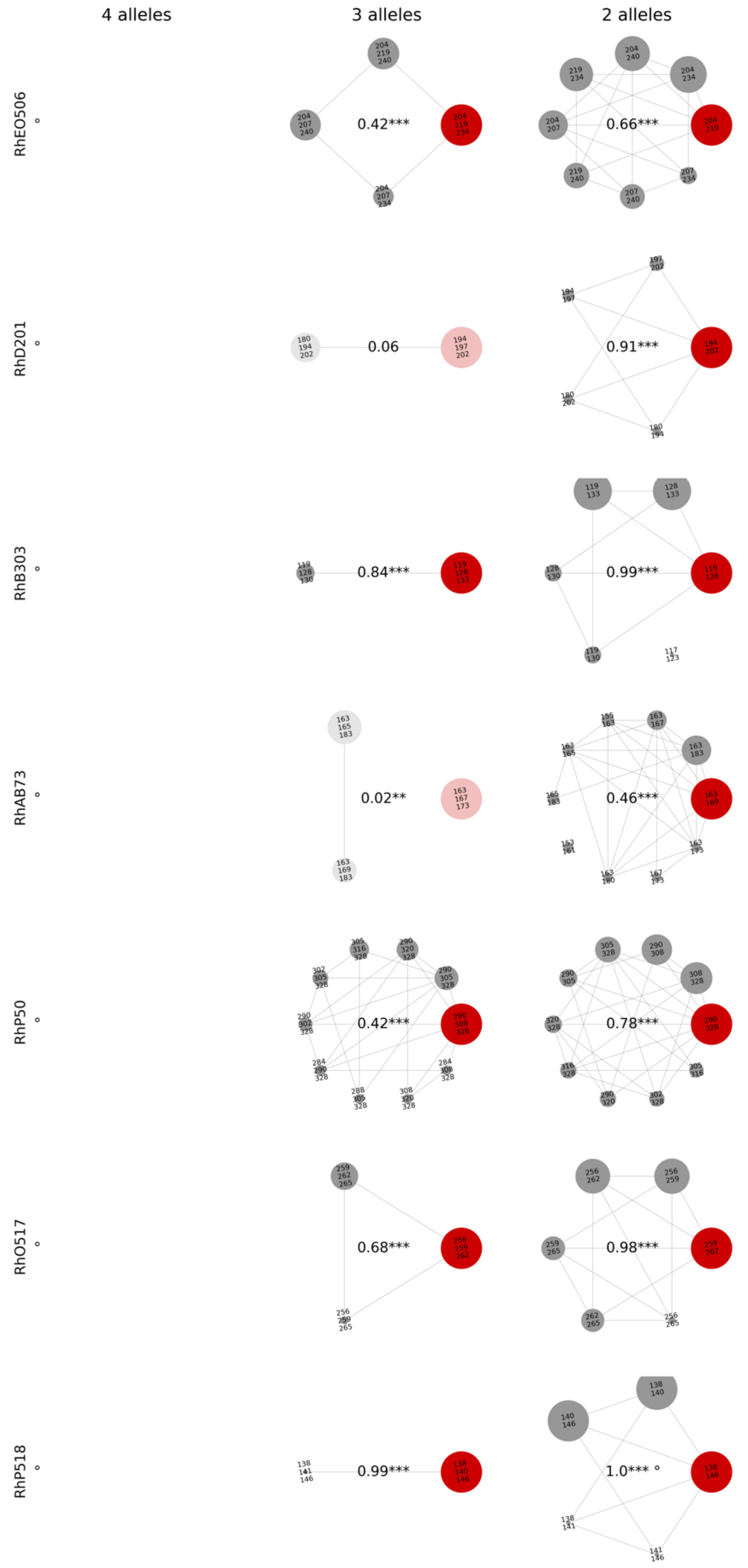

Figure S2-8. Network plots for subsection *Vestitae*: presumably tetraploid samples. Allele combinations differing by only one allele are connected by lines. Circle diameter corresponds to the frequency of the respective allele combination, relative to the most frequent combination (MFAC, in red, maximum diameter). The frequency of the MFAC is displayed in the center of the network, marked by stars if significantly higher than random (* p ≤ 0.05, ** p ≤ 0.01, *** p ≤ 0.001), and by a degree sign if significantly higher within the respective subsection (° p ≤ 0.001).

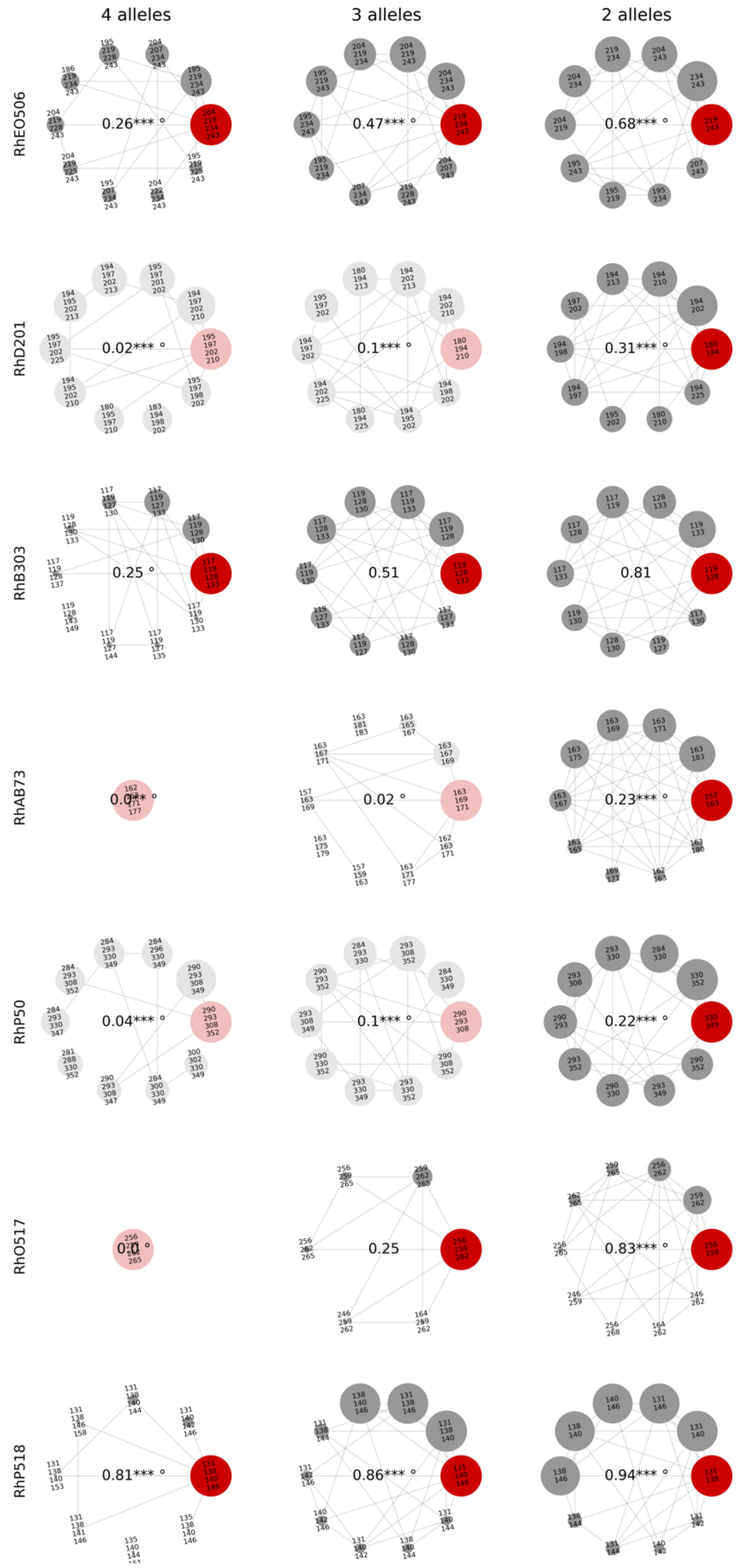

Figure S2-5. Network plots for subsection *Caninae*, one sample per MLG. Allele combinations differing by only one allele are connected by lines. Circle diameter corresponds to the frequency of the respective allele combination, relative to the most frequent combination (MFAC, in red, maximum diameter). The frequency of the MFAC is displayed in the center of the network, marked by stars if significantly higher than random (* p ≤ 0.05, ** p ≤ 0.01, *** p ≤ 0.001), and by a degree sign if significantly higher within the respective subsection (° p ≤ 0.001).

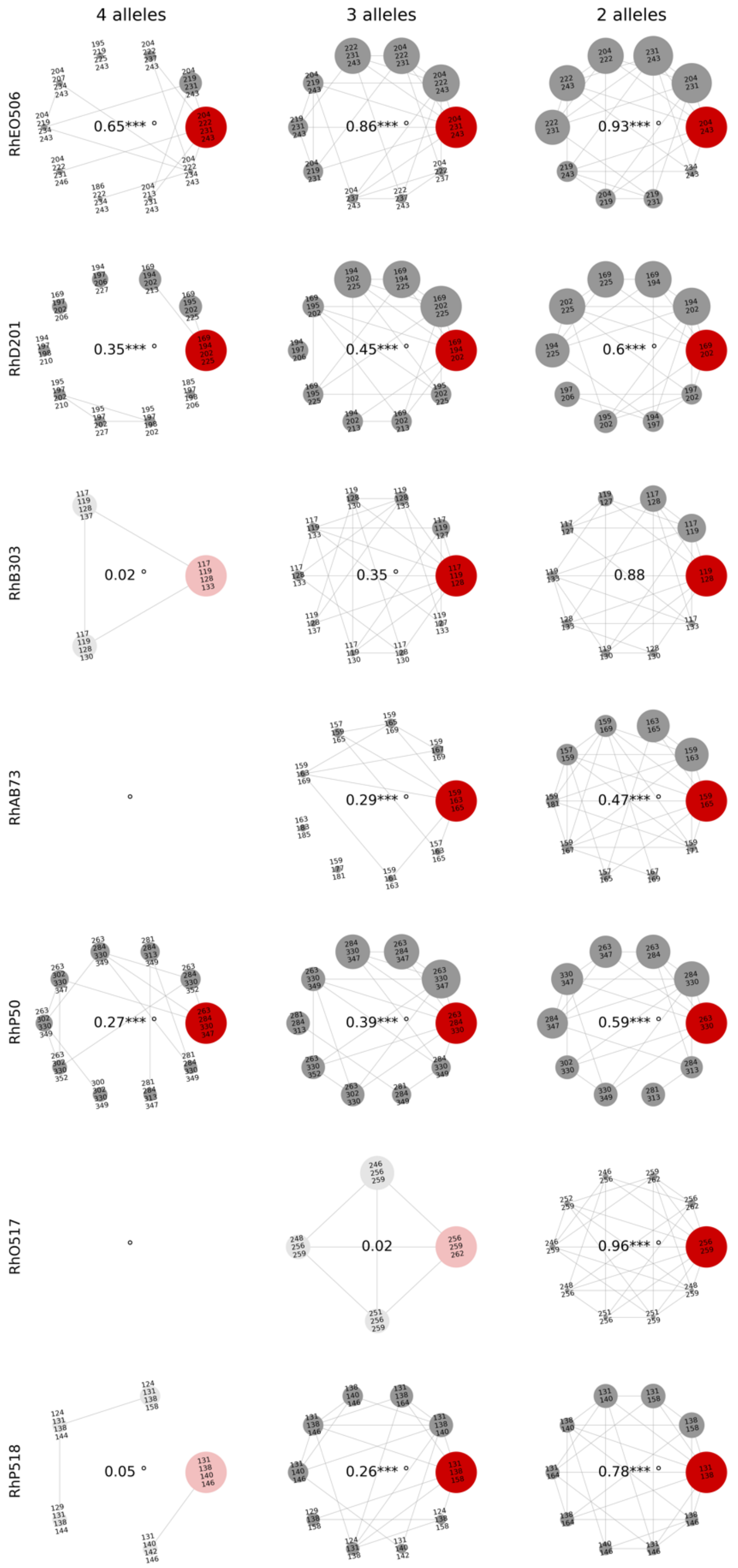

Figure S2-10. Network plots for subsection *Rubigineae*, one sample per MLG. Allele combinations differing by only one allele are connected by lines. Circle diameter corresponds to the frequency of the respective allele combination, relative to the most frequent combination (MFAC, in red, maximum diameter). The frequency of the MFAC is displayed in the center of the network, marked by stars if significantly higher than random (* $p \leq 0.05$, ** $p \leq 0.01$, *** $p \leq 0.001$), and by a degree sign if significantly higher within the respective subsection (° $p \leq 0.001$).

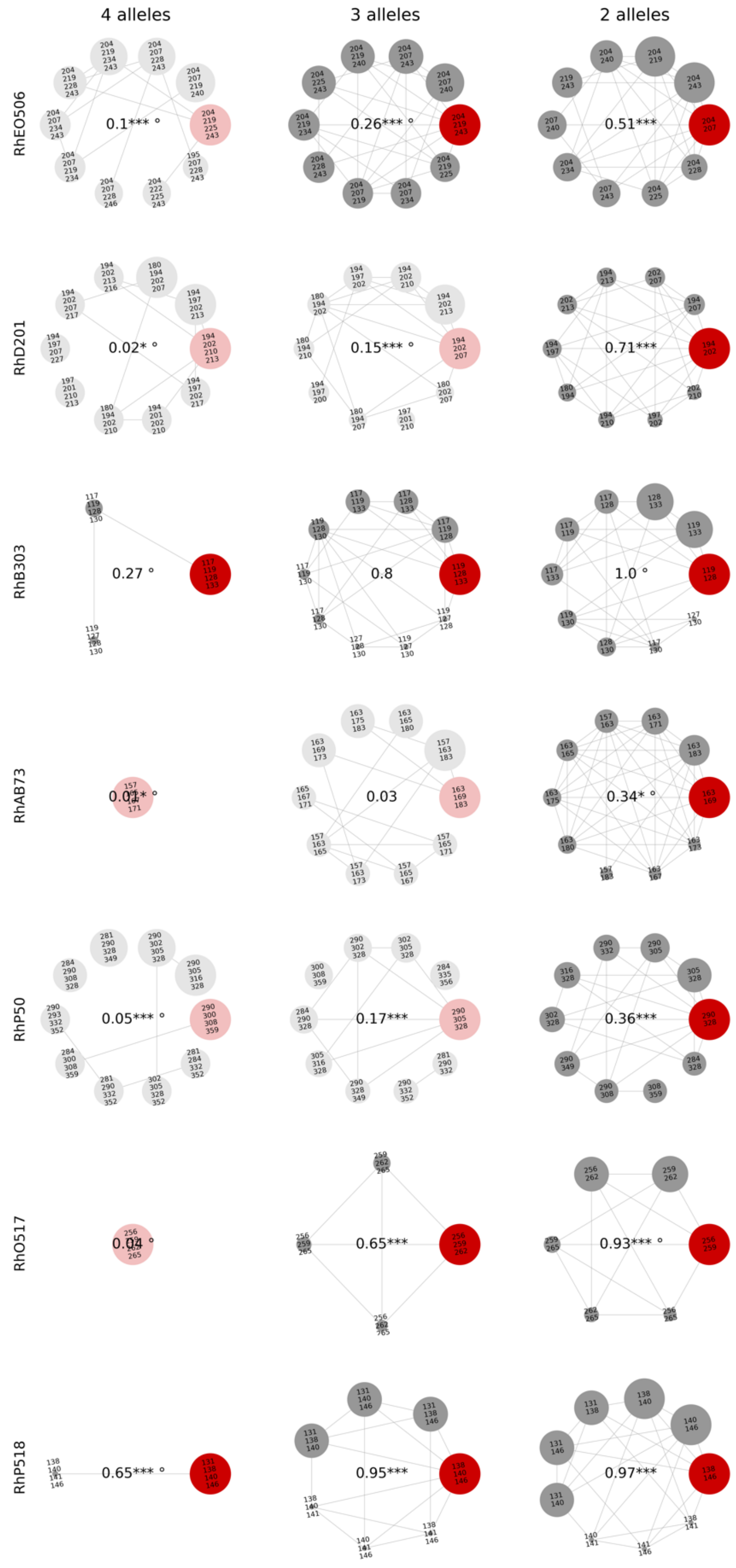

Figure S2-11. Network plots for subsection *Vestitae*: presumably pentaploid samples, one sample per MLG. Allele combinations differing by only one allele are connected by lines. Circle diameter corresponds to the frequency of the respective allele combination, relative to the most frequent combination (MFAC, in red, maximum diameter). The frequency of the MFAC is displayed in the center of the network, marked by stars if significantly higher than random (* $p \leq 0.05$, ** $p \leq 0.01$, *** $p \leq 0.001$), and by a degree sign if significantly higher within the respective subsection (° $p \leq 0.001$).

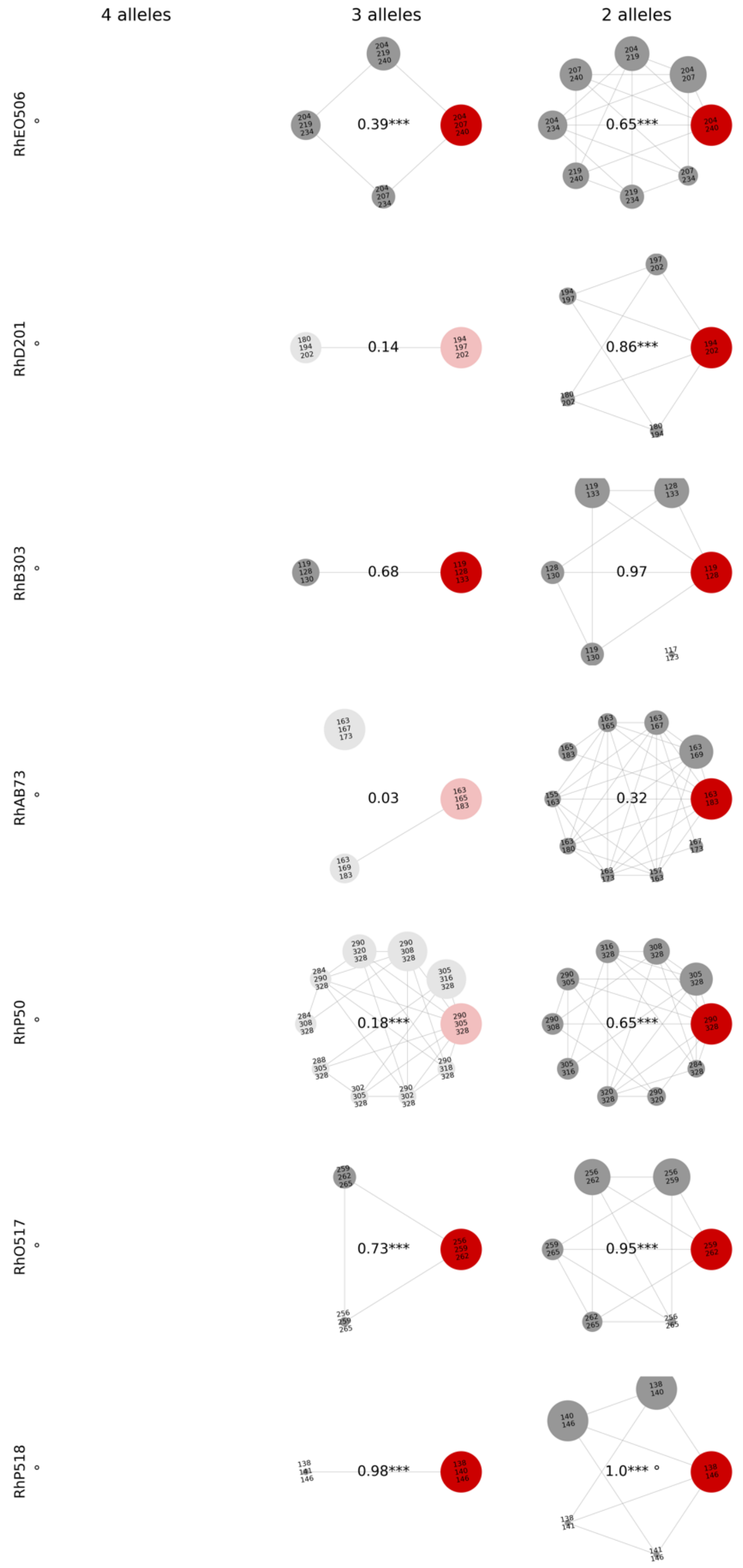

Figure S2-12. Network plots for subsection *Vestitae*: presumably tetraploid samples, one sample per MLG. Allele combinations differing by only one allele are connected by lines. Circle diameter corresponds to the frequency of the respective allele combination, relative to the most frequent combination (MFAC, in red, maximum diameter). The frequency of the MFAC is displayed in the center of the network, marked by stars if significantly higher than random (* $p \leq 0.05$, ** $p \leq 0.01$, *** $p \leq 0.001$), and by a degree sign if significantly higher within the respective subsection (° $p \leq 0.001$).

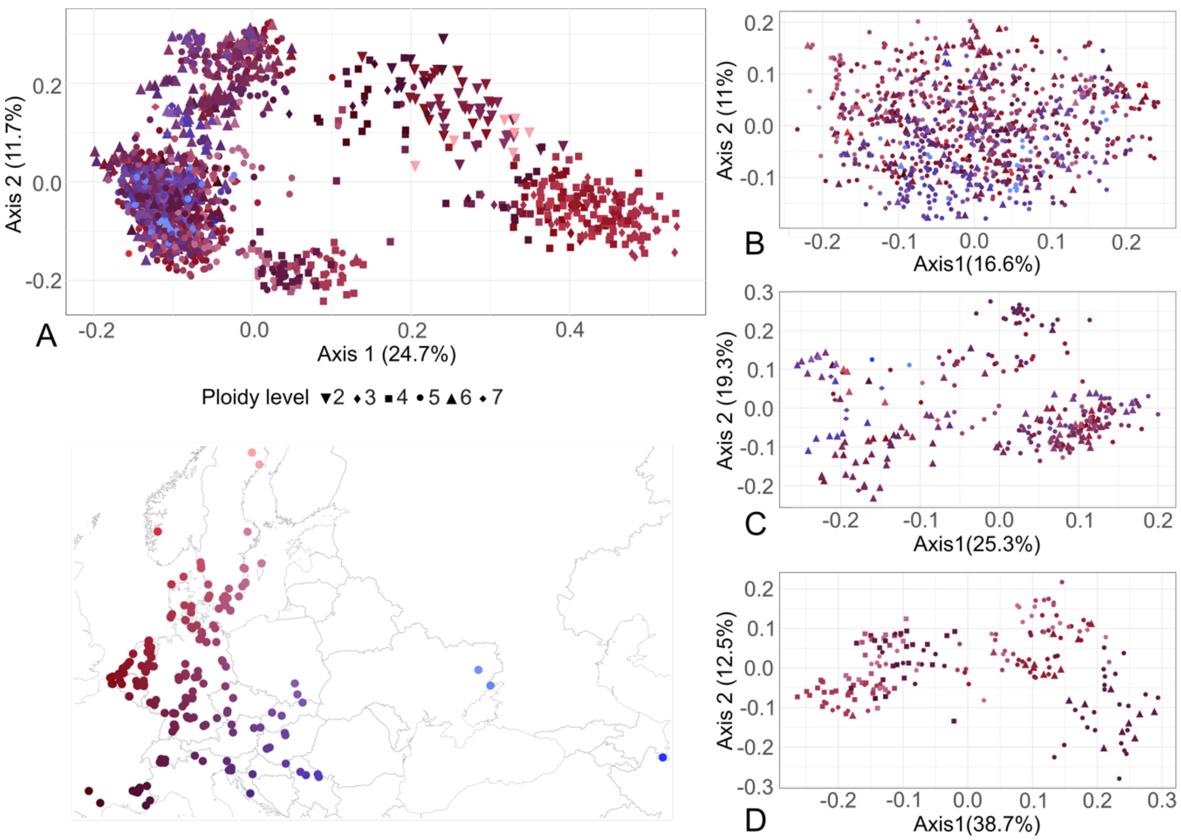

Figure S2-13. PCoA with geographic information. (A) All samples, (B) *Caninae* only, (C) *Rubigineae* only, (D) *Vestitae* only. Color of symbols according to map location of sampling sites; all other parameters identical to Figure 4 in the main text. Country outlines from Natural Earth.
