# Supplementary material for: Untangling the hedge: Genetic diversity in clonally and sexually transmitted genomes of European wild roses, Rosa L
Source: PLoS One. 2023 Oct 5;18(10):e0292634. doi: 10.1371/journal.pone.0292634 (PMC10553836; doi:10.1371/journal.pone.0292634)
Supplement: S3 File — (PDF) [file pone.0292634.s003.pdf]

Reichel et al. 2023 – Genetic Diversity in European roses
Laboratory Methods

Datasets

GE Previously unpublished data produced in the Generose project. The Generose project is described in Van Huylenbroeck et al. (2005)
KW Kellner, Ritz & Wissemann (2014); further details on methods in Kellner, Ritz & Wissemann (2012)
HR Herklotz, Mieder, Ritz (2017) & Herklotz & Ritz (2017)

DNA Extraction

GE Qiagen DNeasy plant mini kit
KW Qiagen DNeasy plant mini kit
HR ATMAB protocol according to Dumoulin et al. (1995)

After determining DNA concentration with a spectrophotometer (Nanodrop), all samples were stored at -80°C until further use.

PCR primer sequences and properties

according to Esselink et al. (2003) and Mayland-Quellhorst & Wissemann (2021); all reverse primers pigtailed with GTTT according to Brownstein et al. 1996.
Linkage groups according to Spiller at al. (2011) and Hibrand Saint-Oyant et al. (2018)

**Locus**	**Linkage group**	**Allele size range [bp]** in GE, KW, HR	**Repeat motif**	**Forward primer** fluorescently labelled	**Reverse primer**
RhEO506	2	186-343	(CAG)(CAA)(CAG)	GAA GCC TCA GCA GCA TCC TCA AAT	GTT TCT TCA GTG CCA ACA AGC CCA TTG G
RhD201	1	165-242	(TCT)	GGT ATG CAA ATA AGA GAT ACA GT	GTT TCT TCC TAA CAA ACC CAT TTT GAA AGG G
RhB303	2	83-151	(GA)	CAC TGC AAC AAC CCA ATA GC	GTT TCT TGT CTT CAG CTT AGA CTG TGC TG
RhAB73	7	151-215	(CT)(CA)	GGT TAG ACG GGT GGA AGA AG	GTT TAC TGC CGA TAG AAG TAT TTC ATC A
RhP50	3	225-406	(CGG)	TGA TGA AAT CAT CCG AGT GTC AG	GTT TCA CTT TCA TTG GAA TGC CAG AAT
RhO517	1	164-275	(GAC)	CGG CGA CGA ACA AAT CAG CAT ATC	GTT TCT TTG AAG AAC GAG GCG CAG CGT AA
RhP518	5	119-184	(CAT)CAAT(CAT)	TTC GAT CTC CAT CTG CAA GA	GTT TCT TCT TAT AAT CTA TTA CGA AGG CTG G

Modifications
HR The forward primers were 5' extended with TGT AAA ACG ACG GCC AGT for M13-fluorescent labeling according to Schuelke (2000)

PCR protocol

Modified from Esselink et al. (2003)

**Reagent**	**GE**	**KW**	**HR**
dd H2O	to total	to total	to total
Polymerase buffer	1x	1x	1x
MgCl2	2 mM	2 mM	2 mM
dNTP-Mix	0.2 mM	0.2 mM	0.2 mM
Taq Polymerase	0.5 U	1U	0.05 U
Primer F	0.4 pmol	0.4 pmol	0.7 pmol
Primer R	0.4 pmol	0.4 pmol	0.36 pmol
Template DNA	30 ng	50 ng	30 ng
Total Volume	12.5 µl	25.0 µl	11.0 µl

PCR programme

GE 94°C 2', [95°C 30", 50°C 30", 72°C 45"]*30, 72°C 5', 4°C ∞
KW 94°C 3', [94°C 30", 53°C 30", 72°C 45"]*28, 72°C 5', 4°C ∞
HR 94°C 3', [94°C 30", 53°C 30", 72°C 45"]*32, 72°C 5', 4°C ∞

Pooling and fluorescent labels

**Locus**	**GE**	**KW**	**HR**
RhEO506	Pool 2 – 6-FAM	6-FAM	6-FAM
RhD201	Pool 1 – 6-FAM	6-FAM	VIC
RhB303	Pool 2 – HEX	HEX	NED
RhAB73	Pool 1 – NED	NED	PET
RhP50	Pool 3 – 6-FAM	6-FAM	VIC
RhO517	Pool 2 – NED	NED	NED
RhP518	Pool 4 – 6-FAM	6-FAM	VIC
		pools variable	pools variable

Fragment Sizing and Scoring

	**Fluorochromes**	**Size standard**	**Sequencer**	**Software**	
**GE**	6-FAM, HEX, NED	GenScan-500 ROX	ABI Prism 3700	Genotyper 3.5 NT	Applied Biosystems
**KW**	6-FAM, HEX, NED	GenScan-500 ROX	ABI 3130 XL	Peak Scanner 1.0	Life Technologies
**HR**	6-FAM, VIC, PET, NE[illegible]	LIZ-500	ABI 3730	Peak Scanner 1.0	Thermo Fisher Scientific

From the measured lengths of M13-labelled fragments, 18 bp were substracted before analysis to account for the added M13 sequence.
When the GE dataset was generated, only three fluorochromes were available for the ABI sequencer.
Note that Thermo Fischer Scientific is the current name for the three companies mentioned here.

Literature

Brownstein ML, Carpten JD, Smith JR (1996): Modulation of non-templated nucleotide addition by Taq DNA polymerase: primer modifications that facilitate genotyping. BioTechniques 20:1004–1010 https://doi.org/10.2144/96206st01

Dumolin S, Demesure B, Petit RJ (1995): Inheritance of chloroplast and mitochondrial genomes in pedunculate oak investigated with an efficient PCR method. Theoretical and Applied Genetics 91: 1253–1256 https://doi.org/10.1007/BF00220937

Esselink D, Smulders MJM, Vosman B (2003): Identification of cut-rose (*Rosa hybrida*) and rootstock varieties using robust Sequence Tagged Microsatellite markers. Theoretical and Applied Genetics 106: 277-286. https://doi.org/10.1007/s00122-002-1122-y

Herklotz V, Mieder N, Ritz CM (2017) Cytological, genetic and morphological variation in mixed stands of dogroses (*Rosa* L. sect. *Caninae* (DC.) Ser.) in Germany with a focus on the hybridogenic *R. micrantha* Sm. Botanical Journal of the Linnean Society. 184:254-71. https://doi.org/10.1093/botlinnean/box025

Herklotz V, Ritz CM (2017) Multiple and asymmetric origin of polyploid dogrose hybrids (*Rosa* L. sect. *Caninae* (DC.) Ser.) involving unreduced gametes. Annals of Botany. 120:209-20. doi: https://doi.org/10.1093/aob/mcw217

Hibrand Saint-Oyant L , Ruttink T, Hamama L, Kirov I, Lakhwani D, Zhou N-N, et al. (2018) A high-quality genome sequence of *Rosa chinensis* to elucidate ornamental traits. Nature Plants 4: 473-484. https://doi.org/10.1038/s41477-018-0166-1

Kellner A, Ritz CM, Wissemann V (2012) Hybridization with invasive *Rosa rugosa* threatens the genetic integrity of native *Rosa mollis.* Botanical Journal of the Linnean Society. 170(3):472-84. https://doi.org/10.1111/j.1095-8339.2012.01298.x

Kellner A, Ritz CM, Wissemann V (2014) Low genetic and morphological differentiation in the European species complex of *Rosa sherardii, R. mollis* and *R. villosa* (*Rosa* section *Caninae* subsection *Vestitae*). Botanical Journal of the Linnean Society. 174(2):240-56. https://doi.org/10.1111/boj.12124.

Mayland-Quellhorst E and Wissemann V (2021): Population structure of *Rosa spinosissima* L. on the Frisian Islands and introgression from cultivated material. Nordic Journal of Botany, 39: e02941. https://doi.org/10.1111/njb.02941

Schuelke M (2000): An economic method for the fluorescent labeling of PCR fragments. Nature Biotechnology 18: 233–234 https://doi.org/10.1038/72708

Spiller M, Linde M, Hibrand-Saint Oyant L, Tsai C-J, Byrne DH, Smulders MJM, Foucher F, Debener T (2011) Towards a unified genetic map of diploid rose. Theoretical and Applied Genetics 122: 489-500. https://doi.org/10.1007/s00122-010-1463-x

Van Huylenbroeck J, Smulders MJM, Debener T, Nybom H, Gudin S, Cox P, et al. (2005) GENEROSE: Genetic evaluation of European rose resources for conservation and horticultural use. Acta Horticulturae (ISHS) 690: 119-124. https://doi.org/10.17660/ActaHortic.2005.690.17
